# Liquid biopsies for cancer: From bench to clinic

**DOI:** 10.1002/mco2.329

**Published:** 2023-07-23

**Authors:** Zhenhui Chen, Chenghao Li, Yue Zhou, Yinghao Yao, Jiaqi Liu, Min Wu, Jianzhong Su

**Affiliations:** ^1^ School of Biomedical Engineering School of Ophthalmology & Optometry and Eye Hospital Wenzhou Medical University Wenzhou Zhejiang China; ^2^ Oujiang Laboratory Zhejiang Lab for Regenerative Medicine Vision and Brain Health Wenzhou Zhejiang China; ^3^ State Key Laboratory of Molecular Oncology National Cancer Center/National Clinical Research Center for Cancer/Cancer Hospital Chinese Academy of Medical Sciences and Peking Union Medical College Beijing China; ^4^ Wenzhou Institute University of Chinese Academy of Sciences Wenzhou Zhejiang China

**Keywords:** cancer, clinical values, epigenetic, liquid biopsy

## Abstract

Over the past two decades, liquid biopsy has been increasingly used as a supplement, or even, a replacement to the traditional biopsy in clinical oncological practice, due to its noninvasive and early detectable properties. The detections can be based on a variety of features extracted from tumor‑derived entities, such as quantitative alterations, genetic changes, and epigenetic aberrations, and so on. So far, the clinical applications of cancer liquid biopsy mainly aimed at two aspects, prediction (early diagnosis, prognosis and recurrent evaluation, therapeutic response monitoring, etc.) and intervention. In spite of the rapid development and great contributions achieved, cancer liquid biopsy is still a field under investigation and deserves more clinical practice. To better open up future work, here we systematically reviewed and compared the latest progress of the most widely recognized circulating components, including circulating tumor cells, cell‐free circulating DNA, noncoding RNA, and nucleosomes, from their discovery histories to clinical values. According to the features applied, we particularly divided the contents into two parts, beyond epigenetics and epigenetic‐based. The latter was considered as the highlight along with a brief overview of the advances in both experimental and bioinformatic approaches, due to its unique advantages and relatively lack of documentation.

## Graphical abstract

Liquid biopsy has been increasingly used as a supplement to the traditional biopsy in clinical oncological practice. This review systematically summarizes the discovery histories, features, and clinical values of the key circulating components. The advantages and disadvantages of these circulating markers beyond or based on epigenetics is also discussed.

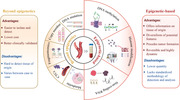

## 1 INTRODUCTION

Comprising a bewildering assortment of diseases, cancer ranks among the top leading causes of death worldwide, killing nearly 10 million people each year.^1^, ^2^ Though the past decades have witnessed an explosion in better understanding of mechanisms underlying tumorigenesis and the development of cancer therapy strategies, it remains a daunting challenge for cancer management, particularly when diagnosed at late stages with poor prognosis.^3^ Thereby there is an urgent need to find efficient tools for early detection and prognostic prediction and to identify accurate biomarkers that allow monitoring of cancer status for long‐lasting surveillance.

Tissue biopsy, which relies on obtaining resected tumor samples through a core needle or open surgery, has long been considered as the gold‐standard method for tumor diagnosis.^4^ Despite its significant contribution, shortcomings do exist, including limitations in early detection and monitoring the ever‐changing complete cancer progression as well as tumor heterogeneity throughout the entire patient journey due to difficulties in repeated specimen acquisition.^4^, ^5^ These snags, have given rise to a new approach: liquid biopsy. By definition, it depends on isolation and analysis of the tumor‑derived entities like circulating tumor cells (CTCs), protein, DNA, and RNA that are presented in bodily fluids such as blood or urine.^6^ Since the complexity and heterogeneity of tumors results from the accumulation of disorders beyond and based on epigenetics, analysis of liquid biopsies has taken into account both these two parts. Specifically, the former usually includes quantitative alterations, somatic mutations, single‐nucleotide variants, and somatic copy‐number aberrations, while epigenetic‐based liquid biopsy includes characterization of DNA methylation, histone posttranslational modification (PTMs), noncoding RNA (ncRNA), and so on.

The feasibility to detect and characterize tumors in such a noninvasive and repeatable way has certainly received tremendous attention, and indeed, liquid biopsy has been demonstrated with considerable clinical implications in tumor diagnosis, prognosis, therapeutic efficacy evaluation, and so on.^7^, ^8^, ^9^ As of April 2023, there are more than 9899 publications listed under the key phrase “cancer liquid biopsies” in PubMed targeting at almost all types of cancers. Meanwhile, more than 3000 clinical trials have been registered at the National Cancer Institute website (http://clinicaltrials.gov). However, the diverse circulating entities, different detection technologies and analysis methods, and complex biological characterizations (e.g., genetic and epigenetic disorders), making it confusing to the cancer research community and clinician. Clear summarizations are always needed to reorganize the massive information for better understanding and utility.

To this end, here, we systemically reviewed the studies and clinical applications of the key components of cancer liquid biopsies (CTCs, circulating tumor DNA [ctDNA], ncRNA, and nucleosomes), along with pointing out the pitfalls and future directions. In particularly, we divided the contents into two parts, beyond epigenetics and epigenetic based. The latter was considered as the highlight due to its immensity clinical potential and relatively lack of documentation. More specifically, we also outlined the recent advances in methodologies for better utility of epigenetic information in liquid biopsy.

## 2 CANCER LIQUID BIOPSY BEYOND EPIGENETICS

Benefiting from the relatively mature techniques, cancer liquid biopsy beyond epigenetics was relatively better documented and clinical applied up so far. In this section, we summarized the biological features of the most representative entities in body fluids, CTCs and ctDNA, and discussed how their disorders beyond epigenetics were used in cancer biopsy.

### 2.1 CTCs biology and clinical values

#### 2.1.1 CTCs biology

CTCs were first discovered in 1869 by Ashworth in the blood of a metastatic cancer patient with appearance similar to those in the primary tumors.^10^ Along with the deepened study, they have been observed in different cancer types such as breast, prostate, lung, and so on, and were found to be presented in various biological fluids in addition to blood, including urine, pleural effusion, ascites, and cerebrospinal fluid.^4^ With that, CTCs were defined nowadays as tumor cells that have been sloughed from either primary or metastatic tumors during an intermediate stage of metastasis and shed into the circulatory systems.^11^, ^12^ However, so far, the morphologies of CTCs could not be well‐defined since they may vary between cancer types and developmental stages.^4^, ^13^ Not surprisingly, they also varied in genome, transcriptome, proteome, and metabolome.^13^, ^14^ Besides, CTCs can circulate as singles cells or congregate with parental tumor cells as well as leukocytes, platelets, or endothelial cells, leading to the formation of aggregates with higher tendency of distant metastases.^15^, ^16^ Indeed, it was reported that CTCs clusters appeared to have 23−50 fold enhanced metastatic capacity, along with a shorter half‐life (25–30 min for single cells vs. 6–10 min for clusters) in the circulation.^17^

#### 2.1.2 CTCs clinical values

Since CTCs may be derived from primary tumors and responsible for the formation of new distant metastases, targeting CTCs at different steps of the metastatic cascade could theoretically break off the progression of metastasis. Yet it has taken more than one century to recognize these critical roles of CTCs in cancer metastasis, largely due to difficulties in isolating the very rare CTCs from the massive pool of circulation of patients.^18^ But the good news is, over the past two decades, the rapid advances of technologies such as high‐throughput sequencing, CRISPR/Cas9 editing tools and single‐cell sequencing allow for relatively more efficient isolation of CTCs and facilitate its clinical applications.^19^, ^20^, ^21^ Indeed, as of April 23, 2023, 1147 results were yielded based on the search term "circulating tumor cells" at ClinicalTrials.gov, further reflecting the great interest in clinic of CTCs. Here, we briefly summarized the most prominent CTCs clinical studies and applications, including both cancer prevention and monitoring.

##### CTCs‐targeting strategies for eliminating cancer metastasis

Intravasation of CTCs into the circulation and extravasation at distant sites are the basic steps of the metastatic cascade.^22^ In a preclinical model of breast cancer (BC), Donato et al.^23^ showed that intratumor hypoxia upregulated cell–cell junction, facilitating the formation of CTC clusters with high ability of metastasis. Accordingly, a proangiogenic therapy via EphrinB2 treatment, which typically targeted at the vascular endothelial growth factor pathway, promoted vascularization and tumor growth rate, meanwhile inhibited intratumor hypoxia and intravasation of clustered CTCs, weakening the metastasis formation.^23^ In the follow‐up study, the authors suggested that some of the already available inhibitors such as PLK1 inhibitors could also prevent CTCs intravasation.^24^ Besides, it was reported pharmacological or genetic obstruction of invadopodia formation (e.g., cortactin or N‐WSAP), maturation (e.g., Tks5), or function (e.g., Tks4) abrogated the extravasation of cancer cell and the subsequent formation of metastatic colony in lung and breast metastasis mouse models.^25^, ^26^

Since accumulating evidence has shown that CTC clusters exhibit more robust metastatic properties than single CTCs, dissociation of CTC clusters therefore offers enormous clinical potential in limiting tumor metastasis. For example, inhibition of heparinase either by genetic knockdown or treatment with its inhibitors such as JG6 was sufficiently enough to destroy the cell clusters formation and repress BC metastasis.^27^ It was also found that injection of BC metastasis mouse model with a clinical thrombolytic agent, the urokinase‐type plasminogen activator, could effectively prevent CTC cluster assembly and prolong the overall host survival rate by up to 20% relative to the control animals.^28^ More recently, in combination of BC patients and mouse models, Gkountela et al.^29^ demonstrated that CTC clustering conferred stem‐like features and could be targeted with United States Food and Drug Administration (US FDA)‐approved cardiac glycosides drugs (Na^+^/K^+^ ATPase inhibitors ouabain and digitoxin), which enabled the dissociation of CTC clusters into single cells, leading to metastasis suppression. Based on these results, a proof‐of‐concept clinical trial has been set up to evaluate the effects of cardiac glycosides on disruption of CTC clusters in nine patients with advanced or metastatic BC (NCT03928210). In addition, dissociation of platelet–cancer cell interactions via antiplatelet agents was proved to be another therapeutic option to prevent tumor metastasis.^15^, ^30^ Among them, aspirin and several COX‐1 inhibitors have been used routinely in the clinic for decades to centuries.^31^, ^32^ And several adenosine 5′‐diphosphate receptor (P2Y_12_) antagonists and a thrombin receptor antagonist have also been approved by US FDA.^31^, ^33^ Similarly, targeting the neutrophils–CTCs association, which contributes to cell cycle progression and metastatic potential of CTCs, also provides opportunity for cancer therapeutic intervention.^34^, ^35^

CTCs are characterized by rewiring of metabolism and homeostasis to support their growth and metastatic functions.^36^ Targeting these vulnerabilities might also be particularly interesting for cancer prevention. For instance, antioxidant defense was reported to be essential for CTCs to evade cell death, and it has indeed been proved that oxidative stress could inhibit the distant metastasis of melanoma.^37^, ^38^ Meanwhile, constriction of pyruvate metabolism was shown to be sufficient to impair collagen hydroxylation and ultimately the progression of lung metastases derived from BC in mouse models.^39^ Likewise, Elia et al.^40^ discovered that proline catabolism via proline dehydrogenase was required for metastasis formation of BC cells, and hampering Prodh was adequate to block formation of lung metastases in mouse models without adverse effects on healthy tissue and organ function.

More recently, accumulating studies have pointed out that immune checkpoint regulators such as PD‐L1 and CD47 were frequently expressed on metastatic cells, suggesting a new way to reduce the recurrence and metastasis of malignant tumors by blocking the immune checkpoints on CTCs.^41^, ^42^, ^43^ Dual blockage of two checkpoint inhibitors (e.g., PD‐L1 and CD47) or one immune checkpoint inhibitor in combination with EpCAM or HER2 could further enhance immunotherapy against CTCs compared with single‐agent therapy.^44^, ^45^, ^46^

##### Clinical predictive value of CTCs

Compared with CTCs for disease prevention, usages of CTCs as biomarkers for cancer early diagnosis, prognosis evaluation, and monitoring of therapeutic response were better documented and have been extensively reviewed.

Actually, whether CTC works in cancer early diagnosis is still controversial to date. It is likely that CTCs are very early events in various cancer types, including lung, breast, pancreatic cancer, and so on.^47^, ^48^, ^49^, ^50^, ^51^ One of the most exciting findings was that, all the five patients with positive CTC count among the 168 patients with chronic obstructive pulmonary disease, developed lung cancer (LC) during follow‐up. Notably, CTCs in this study were detected 1∼4 years earlier than radiological signs of computer tomography‐scan screening.^48^ In liver cancer, Kalinich et al.^52^ suggested that in high‐risk populations, the combination of CTC‐scoring assays with the standard serum biomarker α‐fetoprotein (AFP) may offer higher specificity and sensitivity required for hepatocellular carcinoma (HCC) screening, which AFP alone is unable to provide. These proof‐of‐concept results demonstrated the potential of CTCs detection as an early indicator of patients in high risks.

For patients who have been diagnosed with cancers, CTCs can represent as an independent prognostic factor, which offer valuable prognostic and therapeutic response information to help decision‐making during treatment. Most of the studies were based on CTCs count or molecular characteristics, which were performed by the CellSearch system, the only US FDA‐approved technique for CTC detection used clinically.^53^ A great deal of clinical trials is currently ongoing or completed with either positive or negative feedback across numerous cancers. For example, the PRODIGE 17 trial (NCT01443065) reported that change in CTC counts between baseline and day 28 could help to tailor treatment to each individual patient with advanced gastric and esophageal cancer, since it was significantly associated with overall survival and progression‐free survival.^54^ In prostate cancer (PC), given that androgen receptor splice variant 7 (AR‐V7) functions as a very promising biomarker and patients with CTCs AR‐V7(+) metastatic castration‐resistant prostate cancer (CRPC) had a very poor outcome, Armstrong et al.^55^ conducted a multicenter, prospective‐blinded trial PROPHECY (NCT02269982). AR‐V7 detection in CTCs via two blood‐based assays was shown to be independently linked to worse progression‐free and overall survival with enzalutamide or abiraterone; therefore, alternative treatments should be offered to such patients.^55^ On this basis, a phase II study that was designed for the determination of the response of patients with metastatic CRPC and AR‐V7(+) CTCs to cabazitaxel (NCT03050866) is ongoing.^56^ In BC, while the interventional SWOG‐S0500 trial (NCT00382018) failed to show a benefit of CTCs count guided intervention versus physician's choice at disease progression,^57^ the METABREAST STIC CTC randomized clinical trial (NCT01710605) carried out more recently demonstrated that in hormone receptor‐positive, ERBB2‐negative metastatic BC, CTC count could be used as a reliable marker to guide the decision making for the choice between endocrine therapy and chemotherapy as the first‐line treatment.^58^ Nevertheless, since STIC CTC was performed before the results of the first‐line phase III trials on CDK4/6 inhibitors, the conclusions could not be extrapolated to CDK4/6 combination therapy.^59^

In summary, over the last two decades, the biological characteristics of CTCs and clinical value has been widely investigated in most common cancer types, in both localized and metastatic settings. Even though the benefits of CTCs on cancer is not very clear so far according to results of the extensive clinical trials, but just as said by Aceto and coworkers,^60^ the time is coming for translation of this work into clinical practice thanks to the rapid advances in technology.

### 2.2 ctDNA biology and clinical values beyond epigenetics

#### 2.2.1 cfDNA and ctDNA biology

The term cell‐free DNA (cfDNA) refers to a mixture of nucleic acids that are released outside of cells that can be detected within bodily fluids, which was originally identified in 1948 by Mandel et al.^61^ in blood samples collected from healthy individuals. However, it was not until 1977 that Leon and colleagues discovered increased levels of cfDNA in patients with pancreas cancer, leading to the hypothesis that some of them were tumor derived. Those were later termed as ctDNA.^62^, ^63^ Further studies showed that ctDNA could be detected in almost 100% of the bladder, colorectal, and ovarian cancer (OC), over 50% of cases with most cancer types, and as little as 10% of glioma cases.^64^, ^65^, ^66^

The concentration of cfDNA in healthy adults is generally quite low, often less than 10 ng/mL of plasma. In cancer patients, it can be anywhere 50 times higher but the proportion of ctDNA in the background of overall cfDNA is highly variable, ranging from <0.05 to 90%.^67^ Typically, cfDNA is found as double‐stranded fragments of approximately 150−200 base pairs in length, corresponding to the unit size of nucleosome, while ctDNA comprises strands of <145 bp in length. Besides, the short half‐life of ctDNA in the circulation, which was reported to be from 16 min to 2.5 h, making it a very important tool in monitoring the real‐time situation of tumor dynamics.^67^, ^68^

#### 2.2.2 ctDNA clinical values beyond epigenetics

In recent years, the clinical application of ctDNA has been well established in routine practice and gained enormous attention from both academic groups and commercial vendors (Table 1). The current assays are based on either genetic (mutations, copy number, fragmentations) or epigenetic aberrations. In this part, we will focus on the former, while a relatively detailed discussion about promise of epigenetics in liquid biopsies is given in the next section.

**TABLE 1:** Examples of cancer liquid biopsy products.

Year of approval	Company	Test	Cancer type	Function	Basis	Status
2016	Roche Diagnostics	Cobas EGFR mutation Test v2	NSCLC	Companion diagnostic for targeting treatment	Mutations	US FDA approved
2019	Qiagen Manchester	therascreen PIK3CA RGQ PCR Kit	Breast cancer	Companion diagnostic for targeting treatment	Mutations	US FDA approved
2020	Guardant Health	Guardant 360 CDx	Pan‐cancer	Companion diagnostic for targeting treatment	Mutations	US FDA approved
2020	Foundation Medicine	FoundationOne Liquid CDx	Pan‐cancer	Companion diagnostic for targeting treatment	Mutations	US FDA approved
2019	Invitae Archer DX	Stratafide	Pan‐cancer	Companion diagnostic for targeting treatment	Mutations	FBDD
2017	Natera	Signatera	Pan‐cancer	Molecular residual disease assay	Mutations	FBDD
2020	Thrive Earlier Detection	CancerSEEK	Pan‐cancer	Early detection	Mutations	FBDD
2012	Gen‐Probe	PROGENSA	Prostate cancer	Early detection	lncRNA	US FDA approved
2016	Epigenomics	Epi proColon	CRC	Early detection	DNA methylation	US FDA approved
2020	Bluestar Genomics	Avantect Pancreatic Cancer Test	Pancreatic cancer	Early detection	5hmC	FBDD
2021	AnchorDX	UriFind	Bladder cancer	Early detection	5mC	FBDD

Abbreviations: CRC, colorectal cancer. Data were derived from https://www.hhs.gov/; FBDD, US FDA Breakthrough Devices Designation; NSCLC, non‐small cell lung cancer.

As the model tumor linking genomic disorders to cancer therapy, ctDNA analysis of non‐small cell lung cancer (NSCLC) have paved the road for the Cobas EGFR mutation Test v2 (Roche Diagnostics), the first ctDNA‐based companion diagnostic test to be approved by US FDA and the European Medicines Agency.^69^, ^70^ It was based on the results of a multicenter, open‐label, randomized, Phase III study (ENSURE), which aimed to compare the efficacy and safety of erlotinib to gemcitabine plus cisplatin as first‐line treatment for stage IIIB/IV EGFR‐mutation positive NSCLC patients.^71^ Currently, the test contributes to the detection of patients with advanced NSCLC whose tumor carries substitutions (G719X) in exon 18, deletions in exon 19, insertions and substitutions (T790M, S768I) in exon 20, and substitutions (L858R, L861Q) in exon 21.^72^ In 2020, another companion diagnostic test, FoundationOne Liquid CDx (FoundationOne), which was also based on the detection of EGFR mutations in plasma ctDNA of NSCLC patients, was approved, but using next‐generation sequencing instead of real‐time PCR. It is worth noting that this test also contains ALK rearrangement and METex14‐based detection of NSCLC, PIK3CA mutations‐based detection of BC, BRCA1, BRCA2, and ATM mutations‐based detection of CRPC, and BRCA1 and BRCA2‐based detection of OC.^73^ Meanwhile, a series other assays used for adjuvant therapy were sprang up, such as Guardant 360 CDx assay (Guardant Health) based on simultaneous mutation detection of 55 tumor genes for pan‐cancer, and therascreen PIK3CA RGQ PCR Kit (QIAGEN Manchester) based on PIK3CA mutations for BC.

Another example should be mentioned is the CancerSEEK platform, the first screening methodology for cancer early detection through assessment of the levels of circulating proteins and mutations in cfDNA.^7^, ^74^ It was reported by Cohen et al.^7^ that the CancerSEEK tests were positive in around 70% of the eight cancer types (breast, lung, ovary, stomach, liver, pancreas, colorectum, esophagus). The sensitivities ranged from 69 to 98% for the detection of the five cancer types (esophagus, stomach, ovary, liver, and pancreas) without screening test available for average‐risk individuals.^7^ As a follow‐up study, an exploratory prospective, interventional study, called DETECT‐A was performed to evaluate the feasibility and safety of CancerSEEK coupled with PET‐CT imaging of 10,006 women not previously known to have cancer. The specificity of CancerSEEK alone was estimated to be 98.9%, and increased to 99.6% when combined with PET‐CT.^75^ CancerSEEK is now commercially available since the corresponding clinical trial (NCT04213326) has already completed, but its approvement by US FDA is still awaited.

For the surveillance of cancer recurrence after surgical resection, the first ctDNA‐based marketed assay is Signatera (Natera), a highly personalized and sensitive molecular residual disease assay. Specifically, it is custom designed for each patient to help them to detect relapse earlier than the standard care methods such as carcinoembryonic antigen (CEA) level measurement and CT imaging, and with more than 98% overall positive predictive value of relapse across multiple solid tumors (colorectal, breast lung, bladder cancer, etc.).^76^, ^77^, ^78^

Despite the contributions achieved, negative comments on these assays do exist. For instance, Fakih et al.^79^ recruited 48 patients with resected CRC to compare the specificity and sensitivity of ctDNA surveillance (Signatera) with measurement of CEA levels, imaging, and combination of imaging with CEA levels as suggested by the National Comprehensive Cancer Network guidelines. Results indicated that ctDNA did not achieve better sensitivity than imaging in detecting recurrence (53.3 vs. 60%). And combination of imaging with CEA measurement even showed greater superiority than ctDNA, with sensitivity of 73.3%.^79^ The findings brought into questions the reliability and safety of ctDNA assay regarding the risk of disease recurrence in CRC and the authors therefore called for additional investigations before the adoption of ctDNA solely in clinical practice. Besides, Sullivan et al.^80^ advised that ctDNA is unreliable to detect somatic gene alterations in gastrointestinal peritoneal carcinomatosis with a very low concordance rate of 18%, according to a retrospective analysis of available panel‐based ctDNA results (Guardant 360 CDx) of patients with gastrointestinal cancers treated between 2015 and 2020.

Overall, in addition to the promising potential, caution is warranted in utilizing ctDNA as a therapeutic decision‐making tool in patients, as the detection of ctDNA can be influenced by multiple factors such as disease location, burden, treatment, and tumor vascularization. More investigations are still needed.

## 3 EPIGENETIC‐BASED CANCER LIQUID BIOPSY

**FIGURE 1:**
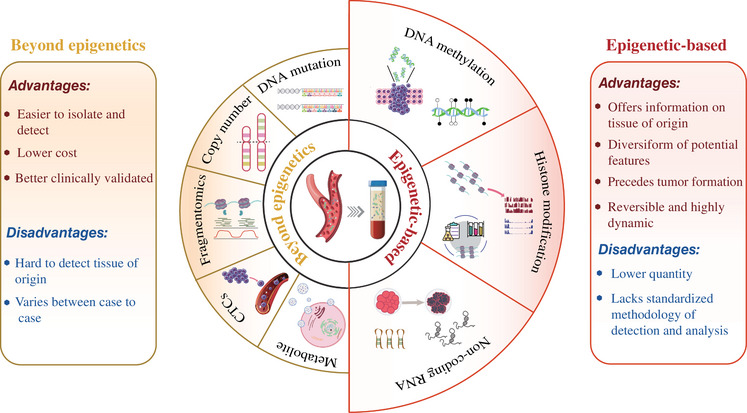
Examples of key circulating components beyond or based on epigenetics. Their advantages and disadvantages are indicated, respectively. The elements were downloaded from https://www.vecteezy.com/ and the figure was created with Adobe Illustrator 2020.

As discussed above, genetic disorders have made great contributions in the field of cancer liquid biopsy. However, it is noteworthy that entities within the circulation of one body share the same genetic information, thus limiting the ability for the detection of tissue‐of‐origin to pinpoint specific cancer type.^1^, ^81^ Meanwhile, genetic information usually varies from case to case, challenging the identification of sensitive and generalizable biomarkers.^82^ Epigenetic profiles could cover perfectly these shortages, as it differs between cells from different tissue types or those with different pathologies but is generalizable across individuals. Even more, the frequency, reversibility, and accessibility of epigenetic alterations in body fluids, endow them the potential to become excellent candidates as clinically valuable cancer biomarkers (Figure 1).^83^, ^84^ In the following sections, we focused particularly on the most widely recognized circulating epigenetic biomarkers, including DNA methylation/hydroxymethylation, histone PTMs, and ncRNAs.

### 3.1 ctDNA methylation and hydroxymethylation

#### 3.1.1 ctDNA 5‐methylcytosine and clinical values

5‐Methylcytosine (5mC) is one of the most deeply studied epigenetic modifications that happens by the addition of a methyl group to the C‐5 position of DNA cytosine ring by DNA methyltransferases, occurring predominantly in CpG dinucleotides.^85^ Cumulative evidence has demonstrated that abnormal DNA methylation plays a critical role in cancer development, mainly through focal hypermethylation at multiple genomic regions (mostly CpG sites), global hypomethylation, or mutagenesis at methylated cytosines.^66^, ^85^, ^86^ Compared with other markers, DNA methylation imbalance typically precedes tumor formation in a high percentage of cancer cases, facilitating early detection.^87^, ^88^ Besides, they are chemically stable and the methylation patterns are often tissue specific, which are helpful to unmask the original primary tumor site of cancer of unknown primary.^89^ These superiorities make cancer‐associated DNA methylation alteration quite attractive for the development of cancer biomarker tests with substantive clinical utility.

**FIGURE 2:**
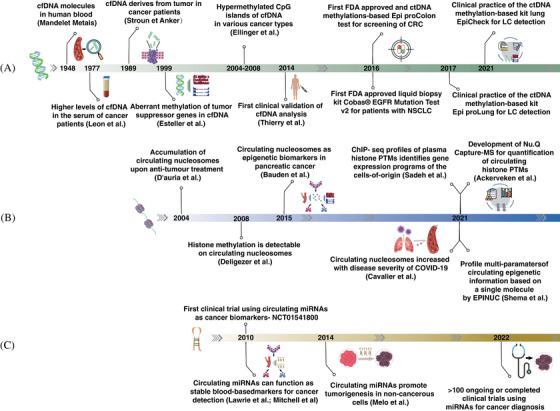
Timeline of main discoveries about epigenetic‐based cancer liquid biopsy with a focus on clinical applications. (A) cfDNA and ctDNA methylation. (B) Circulation nucleosomes and histone PTMs. (C) Circulating miRNA. The elements were downloaded from https://www.vecteezy.com/ and the figure was created with Adobe Illustrator 2020.

The earliest proof that cancer‐related aberrant DNA methylation can be measured in the serum of patients came in 1999, when Wong et al. and Esteller et al. showed promoter hypermethylation of tumor suppressor genes such as p16 in the circulation of HCC and NSCLC patients, respectively (Figure 2).^90^, ^91^ Notwithstanding the limited sample size and number of targeted genes, these two studies that were published back‐to‐back opened the window into taking advantage of DNA methylation profiles in noninvasive detection.^90^, ^91^ Since then, a considerable number of studies have been carried out to characterize changes in methylation of ctDNA, both at the global or gene‐/locus‐ specific level (Table 2). Common hallmarks of various cancers, including global hypomethylation, hypomethylation of DNA repeat elements, oncogene hypomethylation and hypermethylation of CpG islands in the gene promoter, were all demonstrated to be detectable in ctDNA.^84^, ^92^ The identified biomarkers have been proven to provide considerable aid across the whole patient journey from early diagnosis, to treatment and recurrence monitoring (Table 2). A very representative example of ctDNA methylation‐based biomarker is *SEPT9* in colorectal cancer (CRC). Occurring in over 90% of CRC tissues, DNA hypermethylation at the promoter region of *SEPT9* is recognized as a hotspot and is considered to be a specific biomarker of CRC early stages.^93^ In 2008, Sledziewski and coworkers^94^ measured levels of DNA methylation in three marker genes (*SEPT9*, *NGFR*, *TMEFF2*) in human plasma samples from CRC patients or healthy controls, of which *SEPT9* was proved to be the most specific one. After validation by a series of follow‐up case–control studies, the plasma‐based *SEPT9* gene methylation assay, developed as the “Epi proColon” test, was ultimately approved by the US FDA in 2016 as the only blood‐based CRC screening test (Table 1).^66^, ^84^, ^95^ This is an important milestone of bringing circulating methylation biomarkers from the laboratory to the clinical practice. More recently, *SEPT9* was shown to be also effective for CRC postsurgical assessment and prognosis prediction.^96^, ^97^ Soon afterwards, another two commercially available tests for LC detection in plasma‐cfDNA levels namely “Epi proLung” and “lung EpiCheck” were used in clinical practice in 2017 and 2021, respectively. Epi proLung is based on aberrant DNA methylation of stature homeobox 2 (*SHOX2*) and prostaglandin E receptor 4 (*PTGER4*) genes with a fixed specificity of 90% and sensitivity of 67% for LC,^98^ while lung EpiCheck is based on the measurement of methylation level of six genes, enabling detection of up to 85% of early‐stage LC with a specificity of 64% in the high‐risk population.^99^

**TABLE 2:** Summary of recent representative ctDNA methylation‐based liquid biopsy studies in breast, lung, colorectal, ovarian cancer, and multiple cancer types.

Year	Cancer type	Clinical sample size	Type of measured DNA methylation	Markers identified	Function	References
2022	BC	123 patients, 40 controls.	Global	15 markers	Diagnosis and subtyping	^100^
2022	BC	59 patients, 20 controls.	Gene specific	*ESR1*	Therapeutic efficacy evaluation	^101^
2021	BC	204 patients, 129 controls	Gene specific	26 markers	Diagnosis	^102^
2021	BC	101 patients, 102 controls	Global	10 hypomethylation markers	Diagnosis	^103^
2019	BC	200 patients, 35 controls.	Gene specific	*KLK10*, *SOX17*, *WNT5A*, *MSH2*, *GATA3*	Prognosis	^104^
2018	BC	230 patients, 60 controls	Gene specific	*ESR1*	Therapeutic efficacy evaluation	^105^
2017	BC	31 tissues and 1869 serum samples	Global	Six markers, of which *EFC#93*, was validated for clinical use.	Diagnosis	^106^
2016	BC	56 micro‐dissected breast tissue specimens, 29 blood samples from healthy volunteers	Global & gene specific	12 novel epigenetic markers (*JAK3*, *RASGRF1*, *CPXM1*, *SHF*, *DNM3*, *CAV2*, *HOXA10*, *B3GNT5, ST3GAL6*, *DACH1*, *P2RX3*, and chr8:23572595) for detecting BC and four internal control markers (*CREM*, *GLYATL3*, *ELMOD3*, and *KLF9*).	Diagnosis	^107^
2013	BC	319 patients, 237 controls	Gene specific	*ITIH5, DKK3*, and *RASSF1A*	Diagnosis	^108^
2013	BC	193 patients, 60 controls	Gene specific	*SOX7*	Prognosis	^109^
2022	LC	44 patients, 39 controls	Gene specific	*BCAT1*	Diagnosis	^110^
2022	LC	64 patients, 12 controls	Gene specific	*APC, RASSFIA1, FOXA1, SLFN11, SHOX2*	Prognosis	^111^
2021	LC	91 patients, 60 controls	Gene specific	*KMT2C*	Prognosis	^112^
2021	LC	311 patients, 417 controls	Gene specific	6 hypermethylated markers	Diagnosis	^99^
2019	LC	132 patients, 118 controls	Global	9 hypermethylated markers	Diagnosis	^113^
2017	LC	83 patients, 40 controls	Gene specific	*CDO1*, *HOXA9*, *AJAP1*, *PTGDR*, *UNCX*, and *MARCH11*	Diagnosis and prognosis	^114^
2016	LC	118 patients, 212 controls	Gene specific	*SHOX2, PTGER4*	Diagnosis	^98^
2014	LC	122 patients, 24 controls	Gene specific	*BRMS1*	Prognosis	^115^
2022	CRC	15 patients, 5 controls	Global	*CIMP* subtypes	Diagnosis and prognosis	^116^
2022	CRC	55 patients	Gene specific	*SFRP2* and *SDC2*	Therapeutic efficacy evaluation	^117^
2021	CRC	272 patients, 402 controls	Gene specific	*MYO1‐G*	Diagnosis and therapeutic efficacy evaluation	^118^
2021	CRC	96 patients, 78 controls	Gene specific	10 markers	Therapeutic efficacy evaluation	^119^
2020	CRC	801 patients, 1021 controls	Global & gene specific	cg10673833	Diagnosis and prognosis	^120^
2019	CRC	147 patients, 136 controls	Global	Dozens of DNA hypermethylation markers including known (e.g., *SEPT9* and *IKZF1*) and novel (e.g., *EMBP1, KCNQ5, CHST11, APBB1IP*, and *TJP2*) genes	Diagnosis	^121^
2018	CRC	184 patients, 224 controls	Global	*SEPT9* and *SHOX2*	Diagnosis and therapeutic efficacy evaluation	^122^
2018	CRC	182 patients, 50 controls	Global & gene specific	*EYA4*, *GRIA4*, *ITGA4*, *MAP3K14‐AS1*, *MSC*	Therapeutic efficacy evaluation	^123^
2014	CRC	3078 patients, 3796 controls	Gene specific	*SEPT9*	Diagnosis	^124^
2022	OC	249 patients, 288 controls	Global	15 markers	Diagnosis and prognosis	^125^
2020	OC	85 patients, 35 controls	Gene specific	*HOXA9, HIC1*	Diagnosis	^126^
2015	OC	114 patients, 80 controls	Gene specific	*RUNX3, TFPI2, OPCML*	Diagnosis	^127^
2021	Multiple	8542 patients, 6712 controls	Global	>100,000 informative methylation regions	Diagnosis	^128^
2020	Multiple	414 patients, 614 controls	Global	10,613 CpG sites across 477 genomic regions	Diagnosis	^129^
2020	Multiple	2482 patients, 4207 controls	Global	>100,000 informative methylation regions	Diagnosis	^130^

Abbreviations: BC, breast cancer; LC, lung cancer; OC, ovarian cancer.

Certainly, studies of ctDNA methylation to evaluate tumor occurrence, progression, and recurrence have spread to other types of cancers, such as OC, BC, PC,^103^, ^125^, ^131^, ^132^, ^133^ and so on. Specifically, higher frequencies of methylated RASSF1A and BRCA1 in blood‐derived DNA from patients with BC than from healthy females were reported repeatedly. Other tumor suppressor genes, such as *DAPK*, *GSTP1*, *RARB*, *APC*, *SFN*, and *ESR1* were also found to be more frequently methylated in BC cases than in controls.^134^, ^135^, ^136^ In OC, well‐described aberrant methylation‐based markers include *BRCA1*, *RASSF1A*, *OPCML*, *HOXA9*, *RUNX3*, *TFPI2*, and so on. A case in point is *HOXA9*, a homeobox gene normally expressed during differentiation of Mullerian ducts into the female reproductive tract, whose promoter hypermethylation has been suggested as a biomarker for noninvasive diagnosis of OC as well as an indicator of disease grade, since it can be detected in most high‐grade serous OC tissue and plasma samples.^88^ Yet it gave a relative unimpressive result when works alone. For optimization, Singh et al.^126^ developed a novel marker panel with combined analysis of *HOXA9* and *HIC1* hypermethylation, which achieved enhanced sensitivity (88.9%) in distinguishing individuals with OC from controls. Besides, *HOXA9* showed potential in informing clinical decision‐making, as detection of *HOXA9* meth‐ctDNA, at baseline and during treatment with the PARP inhibitor veliparib, was strongly associated with patient outcomes in BRCA1/2‐mutated, platinum‐resistant, and intermediate‐resistant OC.^133^

In fact, in addition to hypermethylation at the promoter of specific tumor suppressor genes, the most dominant epigenetic alteration should be global hypomethylation, particularly in the repetitive DNA sequences, which make about half of the human genome.^137^, ^138^ One of the most well studied examples is the long interspersed nuclear element‐1 (LINE‐1) sequences. Based on analysis of LINE‐1 methylation status in the cfDNA from 21 patients with esophageal adenocarcinoma (*n* = 19) or Barrett's esophagus (*n* = 2), Saggioro and coworkers^137^ showed that hypomethylated LINE‐1 sequences were present in EADC cfDNA and correlated with tumor progression. In CRC, the cfDNA LINE‐1 hypomethylation index (LHI) in patients with large tumors (≥6.0 cm), advanced N stage (≥2), and distant metastasis (M1) was statistically significantly higher than other CRC patients. Additionally, either CRC patients at early stage I/II (*n* = 57) or advanced stage III/IV (*n* = 57) had significantly higher LHI than healthy controls (*n* = 53), suggesting the potential of plasma cfDNA LINE‐1 hypomethylation as a novel biomarker for CRC, particularly for early stage detection.^137^ In NSCLC, Azhikina and coworkers^139^ observed a significant difference of LINE‐1 promoter methylation index between cancer patients (*n* = 56) and healthy controls (*n* = 44) (ROC‐curve analysis: *n* = 100, AUC = 0.69, *p* = 0.0012). Besides, in another study, they indicated a tight association between this serum marker and the pathological process of LC, since changes in the LINE‐1 methylation index of cell surface‐bound csb‐criDNA depended on the patient response to chemotherapy.^140^

There were also a series of arrays targeted at multicancers based on either hyper‐ or hypomethylation regions, or in combination of both (Table 2). For example, it was reported that the DNA methylation‐based blood test, PanSeer assay, was able to detect five common cancer types including stomach, esophageal, colorectal, lung, or liver cancer up to four years prior to conventional diagnosis regardless of tissue‐of‐origin in a robust manner.^129^ A larger case–control study, with 6689 participants [2482 cancer (>50 cancer types), 4207 noncancer] and targeting a panel of >100,000 informative methylation regions from plasma cfDNA was performed by the healthcare company, GRAIL. They successfully detected a wide range of cancer types at both nonmetastatic or metastatic stages, with sensitivity and specificity performance closing to the goal of population‐level screening. Two corresponding clinical trials in intended‐use populations are ongoing (NCT02889978, NCT03085888)^130^ and one of the products Galleri has already been commercially used (Table 1).

#### 3.1.2 ctDNA 5hmC and clinical values

With the mediation of ten‐eleven translocation (TET) enzymes, 5mC can be oxidized to 5‐hydroxymethylcytosine (5hmC), and further to 5‐carboxylcytosine and 5‐formylcytosine.^141^, ^142^ 5hmC is the most abundant and stably oxidized product among them,^143^ but when compared with the 8% methylated cytosine in the human genome, number of hydroxymethylated cytosines is really tiny with only 0.5 to 1%.^144^ Meanwhile, it was reported that in contrast to 5mC, 5hmC‐based profiles are relatively more stable and robust, providing better specificities.^145^ Since 5hmC are found to be enriched in enhancers, promoters, and other regulatory elements, it is understandable that changes in 5hmC are associated with changes in gene expression.^146^, ^147^, ^148^ More recently, aberrations of 5hmC have been linked to various cancers, such as malignant melanoma, BC, bladder cancer, and NSCLC, and have been considered as potential cancer biomarkers and therapeutic targets.^149^

The first proof‐of principle global analysis of hydroxymethylome in cfDNA was carried out in 2017 by Quake et al., who developed a low‐input whole‐genome 5hmC sequencing method based on chemical labelling and applied it on cfDNA from 49 patients of seven different cancer types (LC, hepatocellular carcinoma, pancreatic cancer, glioblastoma, gastric cancer, colorectal cancer, BC). Results showed that cell‐free 5hmC display distinct features that could be used to predict cancer types and stages with high accuracy, proposing a new dimension of information to liquid biopsy‐based diagnosis and prognosis.^150^ Subsequently in 2020, they presented a noninvasive early detection study of pancreatic ductal adenocarcinoma (PDAC) based on 5hmC alterations in cfDNA with AUC of 0.92 in discovery dataset (*n* = 79) and 0.92–0.94 in two independent test sets (*n* = 228).^151^ The corresponding product belongs to Bluestar Genomics, a company dedicated to cancer early detection. Besides, just a few days ago, Levy and coworkers^152^ published their latest research about 5hmC‐based early detection of pancreatic cancer using cfDNA. In combination of 5hmC sequencing with machine learning algorithm, they developed a precise, efficient, and scalable assay, which needs only 10 ng of cfDNA, enabling the effective measurement of cancer presence in individuals at high risk for PaC. The results were based on the case–control clinical trial carried out from June 2018 to May 2022 (NCT03869814), in which cancer and noncancer subjects were recruited from 146 sites across the United States. To provide further supporting evidence on clinical performance of the assay, the authors have launched another interventional study by enrolling up to 6500 subjects from multisite and across multiyear (NCT05188586).

Another team must be introduced is Zhang and his colleagues,^153^ who have also been devoted to the field of 5hmC‐related liquid biopsy for many years. In fact, just one month after Quake et al.’s publication in 2017, Zhang et al.^153^ reported their findings that cfDNA biomarkers based on 5hmC were highly predictive for gastric and CRCs and were superior to other conventional cfDNA biomarkers and meanwhile comparable to 5hmC biomarkers from tissue biopsies. Later, they established a 5hmC‐based diagnostic model, which could accurately discriminate early HCC (stage 0/a) from non‐HCC (validation set: area under curve (AUC) = 88.4%; (95% CI 85.8–91.1%)). Meanwhile, the model showed high capacity for distinguishing early HCC from high‐risk subjects, which were with liver cirrhosis or chronic hepatitis B virus infection (validation set: AUC = 84.6%; (95% CI 80.6–88.7%)). Even more surprisingly, this approach showed superior performance over AFP, the most common serum test applied for HCC screening and diagnosis.^148^

### 3.2 Circulating nucleosome and their PTMs

#### 3.2.1 Nucleosome and histone PTMs

As the basic unit of chromatin in eukaryotes, each nucleosome contains an octamer with two copies of each of the core histones H2A, H2B, H3, and H4, joined together by a linker histone H1 with approximately 146 base pairs of DNA wrapped in 1.67 left‐handed superhelical turns around the octamer.^87^ Histone tails are decorated by a plethora of covalent PTMs on distinct sites and even to several degrees (mono‐, di‐, tri‐) at a single residue, including methylation (me), acetylation (ac), ubiquitylation (ub), and so on. Histone PTMs have been extensively proven to play fundamental roles in the regulation of the underlying genes, but their functions are remarkably specific due to the highly diverse types, in which each mark all has its own message to convey and therefore was suggested to serve as an epigenetic code.^154^ For example, acetylation is generally associated with “relaxed” chromatin and activation of gene transcription, while according to the degree of methylation and the residue methylated, methylation can have various functional consequences. For example, H3K27me3 and H3K9me3 are related to gene silencing but H3K4me2 and H3K4me3 are linked to gene activation.^155^

**FIGURE 3:**
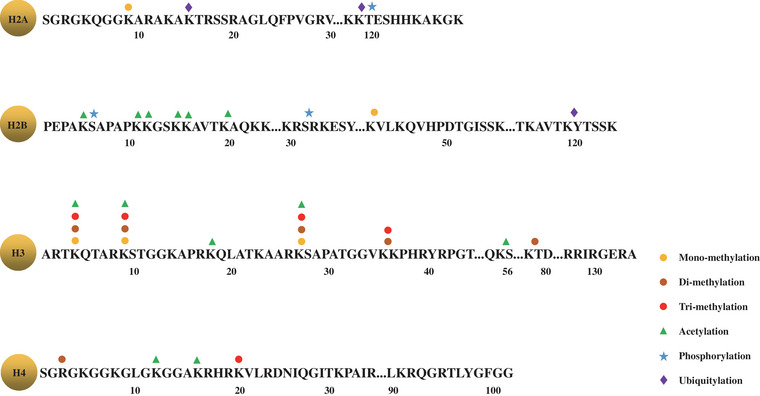
Typical histone posttranslational modifications implicated in cancer. Only canonical histones H2A, H2B, H3, and H4 are involved here. The figure was created with Microsoft Office PowerPoint.

Aberrations of histone PTMs patterns, which are usually a consequence of the deregulation of enzymes responsible for the deposition (known as writers, e.g., histone acetyltransferase, methyltransferase) and removal (known as erasers, e.g., deacetylases, demethylases) of modifications, were identified as general hallmarks of cancer.^156^ The typical histone PTMs as cancer biomarkers are illustrated here in Figure 3. More importantly, PTM players did have shown their excellent capacity as targets of cancer therapy, with several drugs targeting at normalization of H3K27 methylation or histone lysines acetylation having been successfully approved by US FDA.^157^

#### 3.2.2 Circulating histone PTMs and clinical values

Similar to cfDNA, nucleosomes can also be released into biological fluid following cell death or may as well as active mechanisms, which are referred to as circulating nucleosomes.^158^ Actually, it is said some proportion of cfDNA may circulate as nucleosomes or chromosomes, rather than as free DNA.^159^ Since many studies have to be performed retrospectively on serum and plasma samples collected earlier, Sozzi et al.^160^ and Holdenrieder et al.^161^ investigated the loss of DNA and nucleosomes during long‐term storage, respectively. A dramatic decrease of DNA concentration of about 30% per year was reported, while the annual loss of circulating nucleosome is only about 7%, reflecting its relatively more stable structure.^160^, ^161^ Though many studies have demonstrated a generally increased level of circulating nucleosomes in cancers including breast, prostate, colorectal, and LC compared with healthy controls, especially in cases with advanced tumors. However, similar phenomenon can also be observed in many benign diseases, thus reducing their clinical utility for cancer monitoring to a certain extent.^155^

In 2008, Deligezer et al.^162^ found that H3K9me1 was detectable in the promoter region of CDKN2A in a third of the plasma samples from 21 multiple myeloma patients. It was the first study to show evidence of the presence of PTMs in circulating nucleosomes, nearly ten years after the discovery of cfDNA methylation (Figure 2).^162^ Subsequently, a growing number of investigations pointed to the fact that the characterization of PTMs on circulating nucleosomes is an exciting new area that could provide valuable sources for cancer early detection and treatment response monitoring. For instance, CA 19‐9 is regarded as the gold standard marker for PC detection with an average clinical sensitivity of 79 at 82% specificity, while when it was combined with a panel of four epigenetic circulating nucleosome biomarkers (5mC, H2A.Z, H2A1.1, H3K4me2), the sensitivity increased to 92 at 90% specificity.^163^ In CRC, Rahier et al.^164^ suggested an age‐adjusted panel based on three cf‐PTMs (H2AK119Ub, H3K9Ac, H3K27Ac) and the global level of cf‐nucleosomes, which reached an AUC of 0.97 for the distinguish between patients and healthy controls with sensitivity of 75 and 86 at 90% specificity for stages I and II, respectively. More recently, in response to the problem that the concentration of circulating nucleosomes is extremely low but the concentration of native antibodies is high in plasma, Sadeh et al.^165^ devised a protocol for chromatin immunoprecipitation (ChIP)‐seq of cf‐nucleosome from less than 2 mL of plasma and applied it to around 250 samples from more than one hundred subjects, including 61 self‐declared healthy donors, 29 patients suffering from autoimmune, metabolic, or viral liver diseases, 56 patients with metastatic CRC, and four patients with acute myocardial infarction. Levels of four histone PTMs associated with active transcription including H3K4me1/2/3 and H3K36me3 were measured. In addition to the determination of cells of origin, the cfChIP‐seq showed a high sensitivity for detection and identification of subgroups of CRC patients with distinct cancer‐related transcriptional programs.^165^ Later that same year, another study established EPINUC as a novel liquid biopsy approach, allowing analysis of multiple histone PTMs and DNA modifications at single‐molecule precision, which could distinguish CRC patients from healthy controls at high sensitivity and specificity, even at early stages.^166^

It is worth mentioning that the prominent advantage of circulating nucleosomes as biomarkers is the diversiform variety of potential epigenetic features available, which enables fine‐tuning of sensitivity and specificity. Indeed, when assessed individually, the ability of these epigenetic markers in discriminating cancer patients from controls seemed always unremarkable. However, the performance was substantially improved upon combination of multimarkers. Even though neither of these reported panels has been put into clinical practice up to now due to the relatively later starting, a breakthrough is clearly foreseeable in the near future.

### 3.3 Circulating ncRNA

Asides from DNA and proteins, another encouraging source of epigenetic‐based liquid biopsy biomarkers is RNA. In human, approximately 80% of transcripts are noncoding (ncRNA), but functional.^167^ It has been extensively reported that ncRNAs are frequently overexpressed in cancers and suitable for the monitoring of cancer progression as well as recurrence.^168^, ^169^ Accordingly, a series of investigations have suggested the utility of circulating ncRNAs for cancer detection, especially microRNAs (miRNAs) and long ncRNAs (lncRNAs).^170^, ^171^

#### 3.3.1 Circulating miRNA and clinical values

miRNAs are a class of endogenous, single‐stranded, small ncRNAs with an average length of 22 nucleotides. Ever since their discovery in 1993, miRNA was closely watched due to its pivotal role in the gene regulatory network at the posttranscription level.^172^, ^173^ Mechanically, miRNAs function via base pairing to complementary sequences within the target mRNA molecules, resulting in gene silencing by mRNA cleavage or translational repression depending on the degree of complementarity.^174^ As a single miRNA could target up to 400 different mRNAs, it is predicted that more than 30% of the human genes could be directly regulated by miRNAs, involving in almost all fundamental cell processes. Not surprisingly, dysregulations of miRNAs are recognized as features of many pathological processes including cancer.^175^ In general, those significantly overexpressed miRNAs in cancer patients, which accelerate tumor occurrence, development, or metastasis, were considered as oncogenes, and those decreased were regarded as tumor suppressors.^176^ Furthermore, the expression of miRNAs is usually tissue and disease specific, making them potential promising biomarker candidates.

While a majority of miRNAs remain intracellular, about 10% of the known human miRNAs can be secreted into extracellular space and transported to the circulating body fluid such as peripheral blood, which are termed as circulating miRNAs or cell‐free miRNAs. One mind‐blowing point is that unlike cfDNA or circulating nucleosomes, these miRNAs can be easily detected in plasma or serum in a fairly stable form, thanks to the protection from the extracellular environment by binding to special lipid proteins and encapsulation by extracellular vesicles, therefore being resistant to RNase digestions.^176^, ^177^ The half‐life of circulating miRNA is reported to be varied widely between miRNAs, with an average of up to 12 h.^178^

The significance of circulating miRNAs in cancer was first highlighted in 2008 by Lawrie et al.,^179^ who performed a retrospective study to compare levels of tumor‐associated miRNAs (miR‐155, miR‐210, and miR‐21) from the serum of healthy controls and patients with diffuse large B‐cell lymphoma (DLBCL). Both of them were found to be elevated in DLBCL, among which miR‐21 high expression was associated with relapse‐free survival.^179^ Soon afterwards, Mitchell et al.^180^ indicated that serum levels of miR‐141 can robustly discriminate patients with PC from the healthy controls. In the next few years, there was a burst of studies, correlating circulating miRNAs to multiple cancers including breast, bladder, pancreatic, lung, colorectal, and gastric cancers.

Overexpression of miR‐17, miR‐21, miR‐25, miR‐93, miR‐103, miR‐106b, miR‐151, and miR‐181a in serum specimens was shown to be superior to current clinical serum markers in distinguishing early stage esophageal squamous cell carcinoma patients from healthy controls.^181^ Significantly increased signatures of miR‐21, miR‐200a, miR‐200b, miR‐200c, miR‐141, miR‐203, miR‐205, miR‐214, miR‐221, and miR‐622 were observed in the serum of OC with respect to those of the control, as well as decreased miR‐132, miR‐26a, let‐7b, miR‐145, miR‐143, and so on.^182^, ^183^, ^184^ In NSCLC, serum miR‐126, miR‐182, miR183, miR‐210, miR‐19, miR‐20, miR‐21, miR‐125, miR‐145, and miR‐205 were found to possess early detective value, exhibiting similar or even better sensitivity and specificity with traditional tumor marker CEA.^185^, ^186^

Despite its worth in cancer early diagnosis, the utility of circulating miRNA as a tool for segregation of specific cancer subtypes was also well appreciated, which contributes to the determination of tumor mechanisms and selection for more optimized therapeutic approach. Taking the well‐known heterogeneous disease, BC as an example, it can be subclassified according to the presence of estrogen receptor, HER2/neu receptor, and progesterone receptor. Tumors expressing none of these three receptors are named triple‐negative breast cancer (TNBC), which tends to be more advanced and aggressive than other subtypes.^187^ Shin et al. showed that compared with non‐TNBC, the expression of plasma miR‐16, miR‐21, and miR‐199a‐5p was lower in TNBC, among which miR‐199a‐5p expression exhibited the highest value to distinguish TNBC from non‐TNBC and healthy controls.^188^ In contrast, circulating miR‐373 was significantly increased in TNBC than in receptor‐positive tumors.^189^ Another study reported that the serum levels of miR‐16, miR‐155, miR‐21, and miR‐195 not only can discriminate patients with early‐stage BC (stage I or II) from healthy individuals, but also can make an distinguish between those with different molecular subtypes of BC from healthy controls.^190^

Levels of circulating miRNAs also showed a tight association with cancer prognosis and chemotherapy or radiotherapy outcomes. For example, in metastatic CRC, grouping the patients treated with first‐line bevacizumab + chemotherapy based on the changes of circulating miR‐126 disclosed a borderline significant separation of the groups in the progression‐free survival analysis favoring patients with decreasing miR‐126 levels, suggesting that it is worthy of consideration as a possible biomarker for the resistance to anti‐angiogenic containing treatments.^191^ In radiochemotherapy‐treated neck and head squamous cell carcinoma patients, miR‐93‐5p and miR‐425‐5p were identified as the top candidates of commonly deregulated therapy‐responsive circulating miRNAs, but the authors declared that additional independent patient cohort that includes clinical follow‐up data is still required for the further confirmation of the prognostic value of the exciting observation above.^192^

**FIGURE 4:**
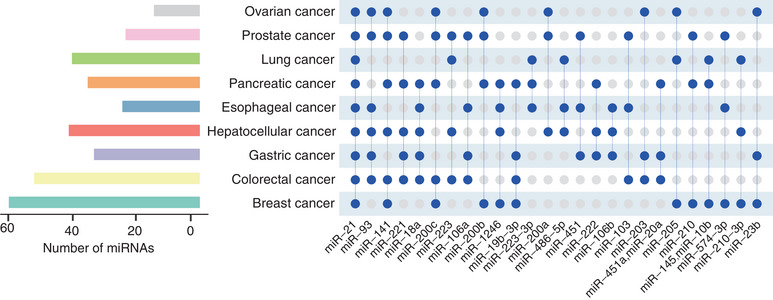
Multicancer‐shared circulating miRNA biomarkers. The list of significantly altered circulating miRNAs in nine common cancers was made to our best knowledge. Error may exist due to incomplete collection. The figure was created with R (version 4.1.0).

The list of circulating miRNA benefits cancer monitoring can go on, we could not cover it all here. Instead, a figure is presented to summarize the frequently altered circulating miRNAs, which have been proven to with diagnostic, prognostic, or predictive relevance in several common human cancers (Figure 4 and Table 3). Of note, the specificity depends on the types of miRNAs to a large extent. For instance, as one of the most abundant and conserved miRNAs, miR‐21 is significantly highly expressed across most cancer types. Interestingly, statistics by Jenike et al.^193^ claimed that miR‐21 has been recognized as a specific predictive or prognostic biomarker by at least 29 diseases; in other words, it has no specificity to any one disease. This surely raises the question that whether it could be considered a viable candidate to be a biomarker for specific cancer, despite its continued evaluation as such.^186^, ^193^

**TABLE 3:** Potential cancer type‐specific circulating miRNA biomarkers in nine common cancers.

Cancer type	Potential cancer type‐specific circulating miRNA biomarkers
Breast cancer	miR‐100‐5p, miR‐106‐3p, miR‐133a, miR‐140‐5p, miR‐148‐3p, miR‐190, miR‐197‐3p, miR‐206, miR‐20b‐5p, miR‐24‐3p, miR‐296, miR‐3160‐5p, miR‐320c, miR‐328‐3p, miR‐331, miR‐34‐5p, miR‐362‐3p, miR‐373, miR‐374a‐5p, miR‐376c‐3p, miR‐4483, miR‐4710, miR‐4755‐3p, miR‐5698, miR‐575, miR‐652‐3p, miR‐8089, miR‐874‐3p, miR‐92a‐3p, miR‐93‐5p, miR‐98‐5p
Colorectal cancer	let‐7b‐3p, miR‐1260b, miR‐1290, miR‐130a, miR‐135, miR‐135b, miR‐139‐3p, miR‐144‐5p, miR‐145‐3p, miR‐150‐3p, miR‐17‐3p, miR‐17‐5p, miR‐18a‐5p, miR‐18b‐5p, miR‐19a, miR‐200b‐3p, miR‐21‐3p, miR‐320d, miR‐338‐5p, miR‐378, miR‐409‐3p, miR‐425‐5p, miR‐4772‐3p, miR‐497, miR‐548c‐5p, miR‐6803‐5p, miR‐7, miR‐720, miR‐92‐3
Gastric cancer	miR‐10b‐5p, miR‐1225‐5p, miR‐143‐3p, miR‐20a‐3p, miR‐217, miR‐296‐5p, miR‐34, miR‐376c, miR‐423‐5p, miR‐501‐3p, miR‐588
Hepatocellular cancer	miR‐122, miR‐122‐5p, miR‐122a, miR‐1247‐3p, miR‐125b, miR‐130b, miR‐142‐3p, miR‐199a, miR‐224, miR‐26a, miR‐34b, miR‐34c, miR‐483, miR‐500, miR‐519d, miR‐548‐2‐3p, miR‐595, miR‐665, miR‐718, miR‐939
Esophageal cancer	miR‐148a‐3p, miR‐151, miR‐20b, miR‐30a‐5p, miR‐339‐5p, miR‐34a‐5p, miR‐584
Pancreatic cancer	miR‐100, miR‐182, miR‐196, miR‐200, miR‐221‐3p, miR‐3679‐5p, miR‐3976, miR‐423‐3p, miR‐4306, miR‐4644, miR‐940
Lung cancer	miR‐125, miR‐1254, miR‐126‐3p, miR‐134, miR‐16‐5p, miR‐181‐5p, miR‐182‐5p, miR‐183‐5p, miR‐185, miR‐19, miR‐198, miR‐20, miR‐22, miR‐23b‐3p, miR‐30a‐3p, miR‐30e‐3p, miR‐320b, miR‐361‐5p, miR‐4257‐3p, miR‐574‐5p, miR‐9‐5p
Prostate cancer	miR‐107, miR‐1845, miR‐26b, miR‐484, miR‐874
Ovarian cancer	miR‐214, miR‐30‐5p

The list of significantly altered circulating miRNAs in nine common cancers was made to our best knowledge. Error may exist due to incomplete collection.

#### 3.3.2 Circulating lncRNA and clinical values

Among the 60,000 ncRNAs in the human genome, >70% are lncRNAs, which comprise a heterogeneous group of transcripts with more than 200 nucleotides in length.^167^ They are located either within intergenic, intronic, antisense stretches or even overlap with protein coding genes.^167^, ^194^ lncRNA has been recognized to be involved in multiple essential biological processes, such as chromatin remodeling, transcriptional and posttranscriptional modification control, and so on.^171^, ^195^, ^196^ In particular, some lncRNAs, such as PVT1, MALAT1, and HOTAIR, were shown to be consistently aberrant in multiple cancers and appear to play key roles in player in carcinogenesis as well as metastasis.^171^

During the past two decades, emerging evidence has indicated that lncRNAs are detectable in various human biofluids and resistant to degradation by ribonucleases, since they are encapsulated inside extracellular vesicles or in association with proteins.^168^ Therefore, similar to miRNA, the consideration of lncRNA as one of the promising sources for noninvasive cancer biomarkers is not surprising. A very successful example is PROGENSA®, the only US FDA‐approved lncRNA used in the diagnostic process for PC based on urine determination of PCA3 (Table 1).^197^ It has to be mentioned that the test took about 14 years from the 1999 basic‐research publication to the 2012 US FDA approval and related‐research continues. For example, Schalken et al.^197^ and Lin et al.^198^ (NCT00756665) indicated that even though PCA3 was shown to be associated with reclassification at sBx1 in a multivariable model, while PCA3, or PCA3 together with TMPRSS2‐ERG, indicated minimal improvement to the clinical utilization of a multivariable model. Therefore, implementing PCA3 and TMPRSS2‐ERG into clinical practice would considerably reduce the number of prostate biopsies.^198^, ^199^, ^200^

Besides, the implication of circulating lncRNA has been extensively studied in many other cancer types. For more information, please refer to the recent reviews that summarized findings regarding lncRNA‐based liquid biopsy, either for several types of cancer^171^, ^195^, ^201^ or focused only on one type of cancer such as NSCLC,^196^ prostate,^202^ gastric,^169^ breast,^170^ DLBCL,^194^ and oral squamous cell carcinoma.^203^

### 3.4 Technical advances for decoding circulating epigenetic information

Despite the growing bodies of work and resources dedicated to this topic, inadequacies still hamper the identification of more reliable promising circulating epigenetic markers to be used as tools to guide cancer prevention and therapy, including: (1) extremely low concordance between different studies; (2) tiny amount in the bloodstream and lack of tools to discriminate those derived from nontumors; (3) a deeper knowledge of the core mechanisms and dynamics of circulating epigenetic markers is needed; (4) conventional analytical and computational strategies are time and resource consuming. Enlarging the cohort size, a more precise patient inclusion criteria, selecting better internal controls, and making discrimination between serum and plasma could be several relatively easier choices to alleviate these issues, nevertheless, to fundamentally figure out relies on the optimization of experimental and analytical methodologies, which we are going to discuss in more detail below.

#### 3.4.1 Experimental techniques

Current methods for the detection of cfDNA methylation can be divided into two categories: or bisulfite conversion‐free or bisulfite conversion‐based methods. Whole‐genome bisulfite sequencing is the most informative and widely‐used cfDNA‐applicable bisulfite‐based one, as it can be performed with 125 pg–30 ng of DNA, but with high prices. Even though methylated CpG tandems amplification and sequencing is more cost friendly and is able to detect hypermethylated CpG sites in ctDNA samples as little as 7.5 pg, its preference for CGGCGG‐rich sites may miss other potential biomarkers.^130^, ^204^ Most importantly, bisulfite treatment is DNA damaging, especially for the tiny amounts of cfDNA, making the detection more challenging. Thus, bisulfite conversion‐free methods based on antibody or magnetic beads enrichment and restriction enzyme were suggested to protect DNA from degradation and adapt to the low cfDNA input. One representative example is TET‐assisted pyridine borane sequencing (TAPS), a newly developed approach based on mild chemistry. Compared with bisulfite sequencing, it improves sequence quality, mapping rate, and coverage while reducing sequencing cost by half. In addition to these advantages in DNA methylation analysis, TAPS is also ideal for simultaneous genetic analysis due to the nondestructive nature.^205^ Indeed, application on HCC and PDAC cfDNA demonstrated that TAPS has the benefit of providing a wealth of information including differential methylation, fragmentation profiles, and tissue of origin, enabling integrated multimodal epigenetic and genetic analysis to accurately discriminate samples from patients with HCC or PDAC from controls. Though the current TAPS protocol has been optimized to work with 10 ng cfDNA purified from 1 to 3 mL of plasma, it remains urgent to lower more the input.^206^

The classical ELISA assay and ChIP followed by quantitative or whole genome sequencing enable gene‐specific or genome‐wide mapping of histone modifications, providing the first glimpse into the rich histone PTMs information present in plasma.^162^, ^163^ However, a large amount of input materials is required while only a single layer of information can be measured, that is, not a cost‐effective way. Sadeh et al.^165^ devised a ChIP‐seq protocol for analysis of cf‐nucleosome from less than 2 mL of plasma to overcome the extremely low concentration of circulating histones as well as the high concentration of native antibodies in plasma, achieving highly specific results that are comparable to the reference ChIP‐seq in tissues. Briefly, ChIP antibodies were covalently immobilized to paramagnetic beads, therefore can be incubated directly in plasma to avoid competition with native antibodies. Meanwhile, prior to the isolation of DNA, the barcoded sequencing‐DNA adaptors are designed to directly ligate to chromatin fragments, forming the so‐called on‐bead adaptor ligation.^165^ Another recently developed system, EPINUC, allows high‐resolution detection and integration of multiple parameters including six active and repressive histone PTMs, their ratios, and combinatorial patterns, from less than 1 mL of plasma sample by single‐molecule imaging, which further expands the burgeoning field of liquid biopsies. Nevertheless, the authors indicated that more studies are still needed to test if this system can make a distinction between different cancer types based solely on the epigenetic profiles.^166^

Circulating ncRNAs are generally considered rather stable, and the related approaches are more sensitive and easier to handle; thus, in my point of view, their extraction and detection are relatively simpler. The most‐used methods are RT‐qPCR and RNA‐Seq. RT‐qPCR is regarded as the gold standard for ncRNA analysis, which offers high sensitivity and high specificity with a large dynamic range, particularly for low RNA input samples, but is only limited to the analysis of known ncRNAs. Instead, RNA‐seq allows for the detection of a larger panel RNA with high sensitivity, including novel ncRNAs. However, relatively time‐consuming protocols, high amount of starting material, and high costs are required.^175^, ^207^

In summary, there is no perfect technology when considering all the tested parameters. Utilization of complementary approaches would make the identification of circulating epigenetic markers more robust.

#### 3.4.2 Computational approaches

It should be pointed out that a single epigenetic biomarker in liquid biopsy usually cannot accurately diagnose or prognosticate cancer due to the heterogeneity of phenotypes and the variability of biomarker expression across individuals.^204^ Integration of multiple parameters based on various aspects of epigenetics as well as mutation‐based, or protein biomarkers, will help to define more robust signatures for specific cancer states. Therefore, there is an unmet need to search for powerful analytical and computational tools for multiparameter analysis since the conventional methods are time and resource consuming, requiring large cohorts and analytical power. Thankfully, the recently rapidly developed machine learning coupled with big data offers an unprecedented opportunity to automatically discover and classify various cancer‐specific signatures from liquid biopsies.^208^

Currently, the traditional machine learning models such as support vector machine (SVM), linear models, and random forest (RF) still account for a major position in early cancer detection due to their training speed and robustness on small dataset.^209^ One of the nice examples was carried out by Bahado‐Singh et al.^210^ on LC. When using plasma CpG biomarkers combined with multiple machine learning algorithms including SVM, RF, generalized linear model, prediction analysis for microarrays, linear discriminant analysis, and deep learning, they achieved highly accurate detection of LC (AUC = 0.90–1.0) with high sensitivity and specificity values.^210^

Meanwhile, algorithms are under updating with leaps and bounds to fill in the information gaps over current methods and further improve the performance. For example, the most‐used DNA methylation analysis focuses on the methylation rate (*β* value) of an individual CpG site in a cell population. Yet, such population‐average measures are shown to be not sensitive enough to capture abnormal methylation signals, which affect only a small number of cfDNAs, thus challenging for the extraction of tiny tumor signals in cfDNA data with low tumor fraction and sequencing coverage. To figure out, Li et al. averaged the methylation values of all CpG sites in a given read and denoted as *α* value. Interestingly, a striking difference (0 and 1) between the normal cfDNAs and abnormally methylated cfDNAs was obtained (*α* tumor = 0% and *α* normal = 100%). They termed this novel, read‐based probabilistic approach as "CancerDetector," which can sensitively identify a trace amount of tumor cfDNAs out of all cfDNAs in plasma.^211^ Nevertheless, the prediction results of CancerDetector can be affected by different depths of sequencing data via introducing systematic deviation, which may reduce the accuracy of cancer diagnosis.^212^ Soon, another approach called DISMIR was proposed, providing ultrasensitive and robust HCC detection, in particularly at low sequencing depths. The outperformance was mainly benefited from the novel design of deep learning model, which combined the DNA sequence together with methylation information for each read, thus grasping sequence motifs related to tumors and extracting the joint patterns of DNA sequence and methylation across different regions from the whole genome to ensure the source prediction of individual reads more accurate. In contrast, the performance of methods based on only methylation information such as RFs, SVM, and FSR was much worse than DISMIR, suggesting the superiority of integrating the information of DNA sequence and methylation.^212^ In addition, Liu et al.^103^ suggested that combining the characteristics of plasma epigenetic signatures with the traditional diagnostic imaging by machine learning could improve the current clinical practice of BC early detection, by reducing the false positive rate and avoiding unnecessary harms.

Rapid technical innovation in the field of epigenetics definitely promotes the development of liquid biopsy, but followed by a waste of the huge amount and multifariousness of data produced from various laboratories measured by different experimental and bioinformatic technologies, because of lacking efficient collection, standardized quality control and analysis procedures. Even though several public databases such as cell‐free epigenome atlas have been introduced to allow easy querying, visualization, and comparison of the collected data by users,^213^ more informative and systematic tools are still needed for efficient integration and reuse of these data.

## 4 CONCLUSIONS AND FUTURE DIRECTIONS

Over the past decades, the emerging field of liquid biopsy has provided exciting new avenues for cancer monitoring and intervention. Indeed, as summarized in this review, there is a growing list of valid circulating indicators, which showed promising ability in clinic or even have been practiced such as Cobas EGFR mutation Test v2, CancerSEEK, Galleri, Epi proColon, and so on. Due to the lack of space, we apologize here to those whose excellent work has not been included. However, to achieve more and better clinical applications, there is still a long and winding road to go, as limitations still exist.

First and foremost, it is unlikely that a single circulating alteration can fulfill all requirements to accurately refine clinical decision‐making; therefore, the combination of different types of biomarkers might provide more reliable results.^83^, ^214^ Cancer liquid biopsy was first introduced from CTCs and rapidly extended to ctDNA and other tumor‐derived products such as circulating nucleosomes, ncRNA, and so on. The features used for discrimination of tumor are also diversifying over the years, from quantitative differences, to genetic disorders and now, plus epigenetic abnormalities. As indicated in Figure 1, each approach has its unique advantages and disadvantages. For instance, CTCs are closely related with cancer metastasis, thus targeting CTCs possesses both the capacities of elimination and prediction of cancer metastasis.^59^, ^60^ While unlike cfDNA or circulating nucleosomes, circulating ncRNAs are usually presented in a fairly stable form, due to the protection by extracellular vesicles.^176^, ^177^ From another dimension of view, though genetic disorders extracted from tumor‑derived entities such as somatic mutations have been relatively well applied in clinic, limitations do exist. For example, entities within the circulation of one body share the same genetic information, but meanwhile varies from individual to individual, challenging the detection of tissue‐of‐origin and identification of generalizable biomarkers.^1^, ^81^ Epigenetic alterations that are reversible, tissue specific, and with higher frequencies thus could nicely balance these shortages.^83^, ^84^ Therefore, since most of the current applications are based on one of the dimensions, it is strongly recommended to make them complement to each other to achieve better performance.

Second, there is a lack of standardized methodology of sampling, isolation, detection and analysis that is sensitive and specific enough to detect sparse circulating biomarkers against the complex substrate of clinical samples, which leads to divergencies between studies and also waste of information source.^204^ At the very beginning, the field focused mainly on development of minimally isolation approaches, which have the capacity to enrich circulating tumor‑derived entities, as they are extremely small in quantity and highly dynamic. As the performance of isolation has improved, it has been driven to the establishment of computational tools for the maximize extraction of useful information. Technological innovation is absolutely the major contributors for promoting clinical practice and should never be prevented, but meanwhile, a motley variety of data was generated from various approaches, which has low mutual compatibilities. To improve data utilization and enhance reliability, it is of great importance to standardize and better organize the existing data, and also establish normative methods for the future work. Besides, as suggested by Keller and Pantel et al.,^18^ development of appropriate reference materials will also contribute to more standardized quality controls, quantification, and reporting among laboratories.

Besides, many studies in the field of cancer liquid biopsy have so far been developed for research or investigational purposes only, since they are lack of standard design of interventional studies and continuous monitoring to demonstrate the clinical utility. Another more important link for the implementation of liquid biopsy from bench to clinical practice is the establishment of evaluation system.

Regarding the assessment of solid tumors in response to traditional cancer therapy, the Response Evaluation Criteria In Solid Tumors (RECIST) provides radiological evaluation as the gold standards.^215^ However, no such guidelines exist to date for the evaluation of liquid biopsy, which is an issue requiring urgent solution. Last, current efforts and clinical practice on cancer liquid biopsy are mainly focused on blood, other bodily fluids such as pleural effusions, urine, and cerebrospinal fluid deserve more attention.

## AUTHOR CONTRIBUTION

J. S., Z. C., and M. W. conceived and designed the study. Z. C., C. L., Y. Z., Y. Y., J. L., and J. S. contributed to the management of data collection and analysis. Z. C., M. W., and J. S. wrote the manuscripts. All authors reviewed and approved the final manuscript.

## CONFLICT OF INTEREST STATEMENT

The authors declare no competing interests.

## ETHICS STATEMENT

Not applicable.

## Data Availability

Not applicable.

## References

[1] Gilbertson RJ . Mapping cancer origins. Cell. 2011;145(1):25‐29. 21458665 10.1016/j.cell.2011.03.019 PMC3077217

[2] Sung H , Ferlay J , Siegel RL , et al. Global Cancer Statistics 2020: GLOBOCAN estimates of incidence and mortality worldwide for 36 cancers in 185 countries. CA Cancer J Clin. 2021;71(3):209‐249. 33538338 10.3322/caac.21660

[3] Badowski C , He B , Garmire LX . Blood‐derived lncRNAs as biomarkers for cancer diagnosis: the good, the bad and the beauty. NPJ Precis Oncol. 2022;6:40. 35729321 10.1038/s41698-022-00283-7 PMC9213432

[4] Hirahata T , R ulQuraish , ul QuraishA , ul Quraish S , Naz M , Razzaq MA . Liquid biopsy: a distinctive approach to the diagnosis and prognosis of cancer. Cancer Inform. 2022;21:11769351221076062. 35153470 10.1177/11769351221076062 PMC8832574

[5] Zhou H , Zhu L , Song J , et al. Liquid biopsy at the frontier of detection, prognosis and progression monitoring in colorectal cancer. Mol Cancer. 2022;21(1):86. 35337361 10.1186/s12943-022-01556-2 PMC8951719

[6] Macías M , Alegre E , Díaz‐Lagares A . Chapter three—Liquid biopsy: from basic research to clinical practice. In: Makowski GS , ed. Advances in Clinical Chemistry. Elsevier; 2018:73‐119. 10.1016/bs.acc.2017.10.003 29304904

[7] Cohen JD , Li L , Wang Y , et al. Detection and localization of surgically resectable cancers with a multi‐analyte blood test. Science. 2018;359(6378):926‐930. 29348365 10.1126/science.aar3247 PMC6080308

[8] Jiang P , Sun K , Peng W , et al. Plasma DNA end‐motif profiling as a fragmentomic marker in cancer, pregnancy, and transplantation. Cancer Discov. 2020;10(5):664‐673. 32111602 10.1158/2159-8290.CD-19-0622

[9] Lone SN , Liquid biopsy: a step closer to transform diagnosis, prognosis and future of cancer treatments. Mol Cancer. 2022. Article number: 79. 10.1186/s12943-022-01543-7 PMC8932066 35303879

[10] Ashworth undefined TR . A case of cancer in which cells similar to those in the tumors were seen in the blood after death. Aust Med J. 1969;14:146‐147.

[11] Zhong X , Zhang H , Zhu Y , et al. Circulating tumor cells in cancer patients: developments and clinical applications for immunotherapy. Mol Cancer. 2020;19:15. 31980023 10.1186/s12943-020-1141-9 PMC6982393

[12] Lin D , Shen L , Luo M , et al. Circulating tumor cells: biology and clinical significance. Signal Transduct Target Ther. 2021;6:404. 34803167 10.1038/s41392-021-00817-8 PMC8606574

[13] Pantel K , Speicher MR . The biology of circulating tumor cells. Oncogene. 2016;35(10):1216‐1224. 26050619 10.1038/onc.2015.192

[14] Micalizzi DS , Maheswaran S , Haber DA . A conduit to metastasis: circulating tumor cell biology. Genes Dev. 2017;31(18):1827‐1840. 29051388 10.1101/gad.305805.117 PMC5695084

[15] Labelle M , Begum S , Hynes RO . Direct signaling between platelets and cancer cells induces an epithelial‐mesenchymal‐like transition and promotes metastasis. Cancer Cell. 2011;20(5):576‐590. 22094253 10.1016/j.ccr.2011.09.009 PMC3487108

[16] Krebs MG , Metcalf RL , Carter L , Brady G , Blackhall FH , Dive C . Molecular analysis of circulating tumour cells—biology and biomarkers. Nat Rev Clin Oncol. 2014;11(3):129‐144. 24445517 10.1038/nrclinonc.2013.253

[17] Aceto N , Bardia A , Miyamoto DT , et al. Circulating tumor cell clusters are oligoclonal precursors of breast cancer metastasis. Cell. 2014;158(5):1110‐1122. 25171411 10.1016/j.cell.2014.07.013 PMC4149753

[18] Keller L , Pantel K . Unravelling tumour heterogeneity by single‐cell profiling of circulating tumour cells. Nat Rev Cancer. 2019;19(10):553‐567. 31455893 10.1038/s41568-019-0180-2

[19] Jones S , dong ChenW , Parmigiani G , et al. Comparative lesion sequencing provides insights into tumor evolution. Proc Natl Acad Sci USA. 2008;105(11):4283‐4288. 18337506 10.1073/pnas.0712345105 PMC2393770

[20] Navin N , Kendall J , Troge J , et al. Tumor evolution inferred by single cell sequencing. Nature. 2011;472(7341):90‐94. 21399628 10.1038/nature09807 PMC4504184

[21] Chen S , Sanjana NE , Zheng K , et al. Genome‐wide CRISPR screen in a mouse model of tumor growth and metastasis. Cell. 2015;160(6):1246‐1260. 25748654 10.1016/j.cell.2015.02.038 PMC4380877

[22] D'Alterio C , Scala S , Sozzi G , Roz L , Bertolini G . Paradoxical effects of chemotherapy on tumor relapse and metastasis promotion. Semin Cancer Biol. 2020;60:351‐361. 31454672 10.1016/j.semcancer.2019.08.019

[23] Donato C , Kunz L , Castro‐Giner F , et al. Hypoxia triggers the intravasation of clustered circulating tumor cells. Cell Rep. 2020;32(10):108105. 32905777 10.1016/j.celrep.2020.108105 PMC7487783

[24] Scheidmann MC , Castro‐Giner F , Strittmatter K , et al. An in vivo CRISPR screen identifies stepwise genetic dependencies of metastatic progression. Cancer Res. 2022;82(4):681‐694. 34916221 10.1158/0008-5472.CAN-21-3908 PMC7612409

[25] Gligorijevic B , Wyckoff J , Yamaguchi H , Wang Y , Roussos ET , Condeelis J . N‐WASP‐mediated invadopodium formation is involved in intravasation and lung metastasis of mammary tumors. J Cell Sci. 2012;125(3):724‐734. 22389406 10.1242/jcs.092726 PMC3367832

[26] Leong HS , Robertson AE , Stoletov K , et al. Invadopodia are required for cancer cell extravasation and are a therapeutic target for metastasis. Cell Rep. 2014;8(5):1558‐1570. 25176655 10.1016/j.celrep.2014.07.050

[27] ru Wei R, i, ni SunD , Yang H , et al. CTC clusters induced by heparanase enhance breast cancer metastasis. Acta Pharmacol Sin. 2018;39(8):1326‐1337. 29417941 10.1038/aps.2017.189 PMC6289387

[28] Choi JW , Kim JK , Yang YJ , Kim P , Yoon KH , Yun SH . Urokinase exerts antimetastatic effects by dissociating clusters of circulating tumor cells. Cancer Res. 2015;75(21):4474‐4482. 26527605 10.1158/0008-5472.CAN-15-0684

[29] Gkountela S , Castro‐Giner F , Szczerba BM , et al. Circulating tumor cell clustering shapes DNA methylation to enable metastasis seeding. Cell. 2019;176(1‐2):98‐112. 30633912 10.1016/j.cell.2018.11.046 PMC6363966

[30] Bambace NM , Holmes CE . The platelet contribution to cancer progression. J Thromb Haemost. 2011;9(2):237‐249. 21040448 10.1111/j.1538-7836.2010.04131.x

[31] Cooke NM , Spillane CD , Sheils O , O'Leary J , Kenny D . Aspirin and P2Y12 inhibition attenuate platelet‐induced ovarian cancer cell invasion. BMC Cancer. 2015;15:627. 26353776 10.1186/s12885-015-1634-x PMC4565001

[32] Xu XR , Yousef GM , Ni H . Cancer and platelet crosstalk: opportunities and challenges for aspirin and other antiplatelet agents. Blood. 2018;131(16):1777‐1789. 29519806 10.1182/blood-2017-05-743187

[33] Zigler M , Kamiya T , Brantley EC , Villares GJ , Bar‐Eli M . PAR‐1 and thrombin: the ties that bind the microenvironment to melanoma metastasis. Cancer Res. 2011;71(21):6561‐6566. 22009534 10.1158/0008-5472.CAN-11-1432 PMC3206157

[34] Szczerba BM , Castro‐Giner F , Vetter M , et al. Neutrophils escort circulating tumour cells to enable cell cycle progression. Nature. 2019;566(7745):553‐557. 30728496 10.1038/s41586-019-0915-y

[35] Masucci MT , Minopoli M , Del Vecchio S , Carriero MV . The emerging role of neutrophil extracellular traps (NETs) in tumor progression and metastasis. Front Immunol. 2020;11:1749. 33042107 10.3389/fimmu.2020.01749 PMC7524869

[36] Elia I , Doglioni G , Fendt SM . Metabolic hallmarks of metastasis formation. Trends Cell Biol. 2018;28(8):673‐684. 29747903 10.1016/j.tcb.2018.04.002

[37] Le Gal K , Ibrahim MX , Wiel C , et al. Antioxidants can increase melanoma metastasis in mice. Sci Transl Med. 2015;7(308). 10.1126/scitranslmed.aad3740 26446958

[38] Piskounova E , Agathocleous M , Murphy MM , et al. Oxidative stress inhibits distant metastasis by human melanoma cells. Nature. 2015;527(7577):186‐191. 26466563 10.1038/nature15726 PMC4644103

[39] Elia I , Rossi M , Stegen S , et al. Breast cancer cells rely on environmental pyruvate to shape the metastatic niche. Nature. 2019;568(7750):117‐121. 30814728 10.1038/s41586-019-0977-x PMC6451642

[40] Elia I , Broekaert D , Christen S , et al. Proline metabolism supports metastasis formation and could be inhibited to selectively target metastasizing cancer cells. Nat Commun. 2017;8:15267. 28492237 10.1038/ncomms15267 PMC5437289

[41] Jilaveanu LB , Shuch B , Zito CR , et al. PD‐L1 expression in clear cell renal cell carcinoma: an analysis of nephrectomy and sites of metastases. J Cancer. 2014;5(3):166‐172. 24563671 10.7150/jca.8167 PMC3931264

[42] Mazel M , Jacot W , Pantel K , et al. Frequent expression of PD‐L1 on circulating breast cancer cells. Mol Oncol. 2015;9(9):1773‐1782. 26093818 10.1016/j.molonc.2015.05.009 PMC5528721

[43] Jaiswal S , Jamieson CHM , Pang WW , et al. CD47 is up‐regulated on circulating hematopoietic stem cells and leukemia cells to avoid phagocytosis. Cell. 2009;138(2):271‐285. 19632178 10.1016/j.cell.2009.05.046 PMC2775564

[44] Müller P , Kreuzaler M , Khan T , et al. Trastuzumab emtansine (T‐DM1) renders HER2+ breast cancer highly susceptible to CTLA‐4/PD‐1 blockade. Sci Transl Med. 2015;7(315):315ra188. 10.1126/scitranslmed.aac4925 26606967

[45] Lian S , Xie R , Ye Y , et al. Dual blockage of both PD‐L1 and CD47 enhances immunotherapy against circulating tumor cells. Sci Rep. 2019;9:4532. 30872703 10.1038/s41598-019-40241-1 PMC6418176

[46] Chen HN , Liang KH , Lai JK , et al. EpCAM signaling promotes tumor progression and protein stability of PD‐L1 through the EGFR pathway. Cancer Res. 2020;80(22):5035‐5050. 32978170 10.1158/0008-5472.CAN-20-1264

[47] Hüsemann Y , Geigl JB , Schubert F , et al. Systemic spread is an early step in breast cancer. Cancer Cell. 2008;13(1):58‐68. 18167340 10.1016/j.ccr.2007.12.003

[48] Ilie M , Hofman V , Long‐Mira E , et al. "Sentinel’’ circulating tumor cells allow early diagnosis of lung cancer in patients with chronic obstructive pulmonary disease. PLoS ONE. 2014;9(10):e111597. 25360587 10.1371/journal.pone.0111597 PMC4216113

[49] Rhim AD , Thege FI , Santana SM , et al. Detection of circulating pancreas epithelial cells in patients with pancreatic cystic lesions. Gastroenterology. 2014;146(3):647‐651. 24333829 10.1053/j.gastro.2013.12.007 PMC4514438

[50] Hosseini H , Obradović MMS , Hoffmann M , et al. Early dissemination seeds metastasis in breast cancer. Nature. 2016;540(7634):552‐558. 27974799 10.1038/nature20785 PMC5390864

[51] Yamaguchi J , Kokuryo T , Yokoyama Y , Ebata T , Ochiai Y , Nagino M . Premalignant pancreatic cells seed stealth metastasis in distant organs in mice. Oncogene. 2021;40(12):2273‐2284. 33649537 10.1038/s41388-021-01706-8

[52] Kalinich M , Bhan I , Kwan TT , et al. An RNA‐based signature enables high specificity detection of circulating tumor cells in hepatocellular carcinoma. Proc Natl Acad Sci USA. 2017;114(5):1123‐1128. 28096363 10.1073/pnas.1617032114 PMC5293050

[53] Cristofanilli M , Budd GT , Ellis MJ , et al. Circulating tumor cells, disease progression, and survival in metastatic breast cancer. N Engl J Med. 2004;351(8):781‐791. 15317891 10.1056/NEJMoa040766

[54] Pernot S , Badoual C , Terme M , et al. Dynamic evaluation of circulating tumour cells in patients with advanced gastric and oesogastric junction adenocarcinoma: prognostic value and early assessment of therapeutic effects. Eur J Cancer. 2017;79:15‐22. 28456090 10.1016/j.ejca.2017.03.036

[55] Armstrong AJ , Halabi S , Luo J , et al. Prospective multicenter validation of androgen receptor splice variant 7 and hormone therapy resistance in high‐risk castration‐resistant prostate cancer: the PROPHECY study. J Clin Oncol. 2019;37(13):1120‐1129. 30865549 10.1200/JCO.18.01731 PMC6494355

[56] Belderbos BPS , Sieuwerts AM , de HoopEO , et al. Associations between AR‐V7 status in circulating tumour cells, circulating tumour cell count and survival in men with metastatic castration‐resistant prostate cancer. Eur J Cancer. 2019;121:48‐54. 31542641 10.1016/j.ejca.2019.08.005

[57] Smerage JB , Barlow WE , Hortobagyi GN , et al. Circulating tumor cells and response to chemotherapy in metastatic breast cancer: sWOG S0500. J Clin Oncol. 2014;32(31):3483‐3489. 24888818 10.1200/JCO.2014.56.2561 PMC4209100

[58] Bidard FC , Jacot W , Kiavue N , et al. Efficacy of circulating tumor cell count–driven vs clinician‐driven first‐line therapy choice in hormone receptor–positive, ERBB2‐negative metastatic breast cancer: the STIC CTC randomized clinical trial. JAMA Oncol. 2021;7(1):34. 33151266 10.1001/jamaoncol.2020.5660 PMC7645742

[59] Vasseur A , Kiavue N , Bidard F , Pierga J , Cabel L . Clinical utility of circulating tumor cells: an update. Mol Oncol. 2021;15(6):1647‐1666. 33289351 10.1002/1878-0261.12869 PMC8169442

[60] Diamantopoulou Z , Castro‐Giner F , Aceto N . Circulating tumor cells: ready for translation? J Exp Med. 2020;217(8):e20200356. 32644115 10.1084/jem.20200356 PMC7398171

[61] Mandel P , Metais P . Nuclear acids in human blood plasma. C R Seances Soc Biol Fil. 1948;142(3‐4):241‐243. 18875018

[62] Leon SA , Shapiro B , Sklaroff DM , Yaros MJ . Free DNA in the serum of cancer patients and the effect of therapy. Cancer Res. 1977;37(3):646‐650. 837366

[63] Oliveira KCS , Ramos IB , Silva JMC , et al. Current perspectives on circulating tumor DNA, precision medicine, and personalized clinical management of cancer. Mol Cancer Res. 2020;18(4):517‐528. 31996469 10.1158/1541-7786.MCR-19-0768

[64] Bettegowda C , Sausen M , Leary RJ , et al. Detection of circulating tumor DNA in early‐ and late‐stage human malignancies. Sci Transl Med. 2014;6(224):224ra24. 10.1126/scitranslmed.3007094 PMC4017867 24553385

[65] Campos‐Carrillo A , Weitzel JN , Sahoo P , et al. Circulating tumor DNA as an early cancer detection tool. Pharmacol Ther. 2019:107458. 31863816 10.1016/j.pharmthera.2019.107458 PMC6957244

[66] Luo H , Wei W , Ye Z , Zheng J , hua XuR . Liquid biopsy of methylation biomarkers in cell‐free DNA. Trends Mol Med. 2021;27(5):482‐500. 33500194 10.1016/j.molmed.2020.12.011

[67] Diehl F , Schmidt K , Choti MA , et al. Circulating mutant DNA to assess tumor dynamics. Nat Med. 2008;14(9):985‐990. 18670422 10.1038/nm.1789 PMC2820391

[68] Corcoran RB , Chabner BA . Application of cell‐free DNA analysis to cancer treatment. N Engl J Med. 2018;379(18):1754‐1765. 30380390 10.1056/NEJMra1706174

[69] Douillard JY , Ostoros G , Cobo M , et al. First‐line gefitinib in Caucasian EGFR mutation‐positive NSCLC patients: a phase‐IV, open‐label, single‐arm study. Br J Cancer. 2014;110(1):55‐62. 24263064 10.1038/bjc.2013.721 PMC3887309

[70] Research C for DE and. cobas EGFR Mutation Test v2. FDA. Published online March 11, 2018. Accessed April 25, 2023. https://www.fda.gov/drugs/resources‐information‐approved‐drugs/cobas‐egfr‐mutation‐test‐v2

[71] Wu YL , Zhou C , Liam CK , et al. First‐line erlotinib versus gemcitabine/cisplatin in patients with advanced EGFR mutation‐positive non‐small‐cell lung cancer: analyses from the phase III, randomized, open‐label, ENSURE study. Ann Oncol. 2015;26(9):1883‐1889. 26105600 10.1093/annonc/mdv270

[72] Kwapisz D . Is it a new era of mutation testing for non‐small cell lung cancer? Ann Transl Med. 2017;5(3):46‐46. The first liquid biopsy test approved. 28251125 10.21037/atm.2017.01.32 PMC5326656

[73] Bayle A , Belcaid L , Aldea M , et al. Clinical utility of circulating tumor DNA sequencing with a large panel: a National Center for Precision Medicine (PRISM) study. Ann Oncol. 2023;34(4):389‐396. 36709039 10.1016/j.annonc.2023.01.008

[74] Markou A , Tzanikou E , Lianidou E . The potential of liquid biopsy in the management of cancer patients. Semin Cancer Biol. 2022;84:69‐79. 35331850 10.1016/j.semcancer.2022.03.013

[75] Lennon AM , Buchanan AH , Kinde I , et al. Feasibility of blood testing combined with PET‐CT to screen for cancer and guide intervention. Science. 2020;369(6499):eabb9601. 32345712 10.1126/science.abb9601 PMC7509949

[76] The TRACERx consortium, The PEACE consortium, Abbosh C , et al. Phylogenetic ctDNA analysis depicts early‐stage lung cancer evolution. Nature. 2017;545(7655):446‐451. 28445469 10.1038/nature22364 PMC5812436

[77] Reinert T , Henriksen TV , Christensen E , et al. Analysis of plasma cell‐free DNA by ultradeep sequencing in patients with stages I to III colorectal cancer. JAMA Oncol. 2019;5(8):1124. 31070691 10.1001/jamaoncol.2019.0528 PMC6512280

[78] Christensen E , Birkenkamp‐Demtröder K , Sethi H , et al. Early detection of metastatic relapse and monitoring of therapeutic efficacy by ultra‐deep sequencing of plasma cell‐free DNA in patients with urothelial bladder carcinoma. J Clin Oncol. 2019;37(18):1547‐1557. 10.1200/JCO.18.02052 31059311

[79] Fakih M , Sandhu J , Wang C , et al. Evaluation of comparative surveillance strategies of circulating tumor DNA, imaging, and carcinoembryonic antigen levels in patients with resected colorectal cancer. JAMA Netw Open. 2022;5(3):e221093. 35258578 10.1001/jamanetworkopen.2022.1093 PMC8905389

[80] Sullivan BG , Lo A , Yu J , et al. Circulating tumor DNA is unreliable to detect somatic gene alterations in gastrointestinal peritoneal carcinomatosis. Ann Surg Oncol. 2023;30(1):278‐284. 35980549 10.1245/s10434-022-12399-y PMC9726669

[81] Lo YMD , Han DSC , Jiang P , Chiu RWK . Epigenetics, fragmentomics, and topology of cell‐free DNA in liquid biopsies. Science. 2021;372(6538):eaaw3616. 33833097 10.1126/science.aaw3616

[82] Gai W , Sun K . Epigenetic biomarkers in cell‐free DNA and applications in liquid biopsy. Genes. 2019;10(1):32. 30634483 10.3390/genes10010032 PMC6356936

[83] Costa‐Pinheiro P , Montezuma D , Henrique R , Jerónimo C . Diagnostic and prognostic epigenetic biomarkers in cancer. Epigenomics. 2015;7(6):1003‐1015. 26479312 10.2217/epi.15.56

[84] Duforestel M , Briand J , Bougras‐Cartron G , et al. Cell‐free circulating epimarks in cancer monitoring. Epigenomics. 2020;12(2):145‐155. 31916450 10.2217/epi-2019-0170

[85] Dor Y , Cedar H. Principles of DNA methylation and their implications for biology and medicine. *Lancet*. 2018;392:10. 10.1016/S0140-6736(18)31268-6 30100054

[86] Flavahan WA , Gaskell E , Bernstein BE . Epigenetic plasticity and the hallmarks of cancer. Science. 2017;357(6348):eaal2380. 28729483 10.1126/science.aal2380 PMC5940341

[87] Kanwal R , Gupta K , Gupta S . Cancer epigenetics: an introduction. In: Verma M , ed. Cancer Epigenetics. Springer; 1238:3‐25. Methods in Molecular Biology. 10.1007/978-1-4939-1804-1_1 25421652

[88] Hentze JL , Høgdall CK , Høgdall EV . Methylation and ovarian cancer: can DNA methylation be of diagnostic use? (Review). Mol Clin Oncol. 2019;10(3):323‐330. 30847169 10.3892/mco.2019.1800 PMC6388465

[89] Moran S , Martínez‐Cardús A , Sayols S , et al. Epigenetic profiling to classify cancer of unknown primary: a multicentre, retrospective analysis. Lancet Oncol. 2016;17(10):1386‐1395. 27575023 10.1016/S1470-2045(16)30297-2

[90] Esteller M , Sanchez‐Cespedes M , Rosell R , Sidransky D , Baylin SB , Herman JG . Detection of aberrant promoter hypermethylation of tumor suppressor genes in serum DNA from non‐small cell lung cancer patients. Cancer Res. 1999;59(1):67‐70. 9892187

[91] Wong IH , Lo YM , Zhang J , et al. Detection of aberrant p16 methylation in the plasma and serum of liver cancer patients. Cancer Res. 1999;59(1):71‐73. 9892188

[92] Nishiyama A , Nakanishi M . Navigating the DNA methylation landscape of cancer. Trends Genet. 2021;37(11):1012‐1027. 34120771 10.1016/j.tig.2021.05.002

[93] Song L , LiY . Advances in Clinical Chemistry. Elsevier; 2015:171‐204. SEPT9. 10.1016/bs.acc.2015.07.004 26471083

[94] Lofton‐Day C , Model F , DeVos T , et al. DNA methylation biomarkers for blood‐based colorectal cancer screening. Clin Chem. 2008;54(2):414‐423. 18089654 10.1373/clinchem.2007.095992

[95] deVos T , Tetzner R , Model F , et al. Circulating methylated SEPT9 DNA in plasma is a biomarker for colorectal cancer. Clin Chem. 2009;55(7):1337‐1346. 19406918 10.1373/clinchem.2008.115808

[96] Fu B , Yan P , Zhang S , et al. Cell‐free circulating methylated SEPT9 for noninvasive diagnosis and monitoring of colorectal cancer. Dis Markers. 2018;2018:6437104. 29849824 10.1155/2018/6437104 PMC5937566

[97] Song L , Guo S , Wang J , et al. The blood mSEPT9 is capable of assessing the surgical therapeutic effect and the prognosis of colorectal cancer. Biomark Med. 2018;12(9):961‐973. 30043648 10.2217/bmm-2018-0012

[98] Weiss G , Schlegel A , Kottwitz D , König T , Tetzner R . Validation of the SHOX2/PTGER4 DNA methylation marker panel for plasma‐based discrimination between patients with malignant and nonmalignant lung disease. J Thorac Oncol. 2017;12(1):77‐84. 27544059 10.1016/j.jtho.2016.08.123 PMC5226366

[99] Gaga M , Chorostowska‐Wynimko J , Horváth I , et al. Validation of Lung EpiCheck, a novel methylation‐based blood assay, for the detection of lung cancer in European and Chinese high‐risk individuals. Eur Respir J. 2021;57(1):2002682. 33122336 10.1183/13993003.02682-2020 PMC7806969

[100] Gao Y , Zhao H , An K , et al. Whole‐genome bisulfite sequencing analysis of circulating tumour DNA for the detection and molecular classification of cancer. Clin Transl Med. 2022;12(8):e1014. 35998020 10.1002/ctm2.1014 PMC9398227

[101] Bos MK , Deger T , Sleijfer S , Martens JWM , Wilting SM . ESR1 methylation measured in cell‐free DNA to evaluate endocrine resistance in metastatic breast cancer patients. Int J Mol Sci. 2022;23(10):5631. 35628441 10.3390/ijms23105631 PMC9142900

[102] Zhang X , Zhao D , Yin Y , et al. Circulating cell‐free DNA‐based methylation patterns for breast cancer diagnosis. NPJ Breast Cancer. 2021;7:106. 34400642 10.1038/s41523-021-00316-7 PMC8367945

[103] Liu J , Zhao H , Huang Y , et al. Genome‐wide cell‐free DNA methylation analyses improve accuracy of non‐invasive diagnostic imaging for early‐stage breast cancer. Mol Cancer. 2021;20:36. 33608029 10.1186/s12943-021-01330-w PMC7893735

[104] Panagopoulou M , Karaglani M , Balgkouranidou I , et al. Circulating cell‐free DNA in breast cancer: size profiling, levels, and methylation patterns lead to prognostic and predictive classifiers. Oncogene. 2019;38(18):3387‐3401. 30643192 10.1038/s41388-018-0660-y

[105] Mastoraki S , Strati A , Tzanikou E , et al. *ESR1* methylation: a liquid biopsy–based epigenetic assay for the follow‐up of patients with metastatic breast cancer receiving endocrine treatment. Clin Cancer Res. 2018;24(6):1500‐1510. 29284708 10.1158/1078-0432.CCR-17-1181

[106] Widschwendter M , Evans I , Jones A , et al. Methylation patterns in serum DNA for early identification of disseminated breast cancer. Genome Med. 2017;9:115. 29268762 10.1186/s13073-017-0499-9 PMC5740791

[107] Uehiro N , Sato F , Pu F , et al. Circulating cell‐free DNA‐based epigenetic assay can detect early breast cancer. Breast Cancer Res BCR. 2016;18:129. 27993161 10.1186/s13058-016-0788-z PMC5168705

[108] Kloten V , Becker B , Winner K , et al. Promoter hypermethylation of the tumor‐suppressor genes ITIH5, DKK3, and RASSF1A as novel biomarkers for blood‐based breast cancer screening. Breast Cancer Res BCR. 2013;15(1):R4. 23320751 10.1186/bcr3375 PMC3672828

[109] Chimonidou M , Strati A , Malamos N , Georgoulias V , Lianidou ES . SOX17 promoter methylation in circulating tumor cells and matched cell‐free DNA isolated from plasma of patients with breast cancer. Clin Chem. 2013;59(1):270‐279. 23136251 10.1373/clinchem.2012.191551

[110] Palanca‐Ballester C , Hervas D , Villalba M , et al. Translation of a tissue epigenetic signature to circulating free DNA suggests BCAT1 as a potential noninvasive diagnostic biomarker for lung cancer. Clin Epigenetics. 2022;14(1):116. 36123616 10.1186/s13148-022-01334-3 PMC9487112

[111] Markou Londra D , Tserpeli V , et al. DNA methylation analysis of tumor suppressor genes in liquid biopsy components of early stage NSCLC: a promising tool for early detection. Clin Epigenetics. 2022;14:61. 35538556 10.1186/s13148-022-01283-x PMC9092693

[112] Mastoraki S , Balgkouranidou I , Tsaroucha E , Klinakis A , Georgoulias V , Lianidou E . KMT2C promoter methylation in plasma‐circulating tumor DNA is a prognostic biomarker in non‐small cell lung cancer. Mol Oncol. 2021;15(9):2412‐2422. 33159839 10.1002/1878-0261.12848 PMC8410531

[113] Liang W , Zhao Y , Huang W , et al. Non‐invasive diagnosis of early‐stage lung cancer using high‐throughput targeted DNA methylation sequencing of circulating tumor DNA (ctDNA). Theranostics. 2019;9(7):2056‐2070. 31037156 10.7150/thno.28119 PMC6485294

[114] Ooki A , Maleki Z , Tsay JCJ , et al. A panel of novel detection and prognostic methylated DNA markers in primary non–small cell lung cancer and serum DNA. Clin Cancer Res. 2017;23(22):7141‐7152. 28855354 10.1158/1078-0432.CCR-17-1222

[115] Balgkouranidou I , Chimonidou M , Milaki G , et al. Breast cancer metastasis suppressor‐1 promoter methylation in cell‐free DNA provides prognostic information in non‐small cell lung cancer. Br J Cancer. 2014;110(8):2054‐2062. 24642624 10.1038/bjc.2014.104 PMC3992488

[116] Cheruba E , Viswanathan R , Wong PM , et al. Heat selection enables highly scalable methylome profiling in cell‐free DNA for noninvasive monitoring of cancer patients. Sci Adv. 2022;8(36):eabn4030. 36083902 10.1126/sciadv.abn4030 PMC9462700

[117] Barták BK , Fodor T , Kalmár A , et al. A liquid biopsy‐based approach for monitoring treatment response in post‐operative colorectal cancer patients. Int J Mol Sci. 2022;23(7):3774. 35409133 10.3390/ijms23073774 PMC8998310

[118] Lin WH , Xiao J , Ye ZY , et al. Circulating tumor DNA methylation marker MYO1‐G for diagnosis and monitoring of colorectal cancer. Clin Epigenetics. 2021;13:232. 34961566 10.1186/s13148-021-01216-0 PMC8713401

[119] Jin S , Zhu D , Shao F , et al. Efficient detection and post‐surgical monitoring of colon cancer with a multi‐marker DNA methylation liquid biopsy. Proc Natl Acad Sci USA. 2021;118(5):e2017421118. 33495330 10.1073/pnas.2017421118 PMC7865146

[120] Luo H , Zhao Q , Wei W , et al. Circulating tumor DNA methylation profiles enable early diagnosis, prognosis prediction, and screening for colorectal cancer. Sci Transl Med. 2020;12(524):eaax7533. 31894106 10.1126/scitranslmed.aax7533

[121] Li J , Zhou X , Liu X , et al. Detection of colorectal cancer in circulating cell‐free DNA by methylated CpG tandem amplification and sequencing. Clin Chem. 2019;65(7):916‐926. 31010820 10.1373/clinchem.2019.301804

[122] Bergheim J , Semaan A , Gevensleben H , et al. Potential of quantitative SEPT9 and SHOX2 methylation in plasmatic circulating cell‐free DNA as auxiliary staging parameter in colorectal cancer: a prospective observational cohort study. Br J Cancer. 2018;118(9):1217‐1228. 29610456 10.1038/s41416-018-0035-8 PMC5943265

[123] Barault L , Amatu A , Siravegna G , et al. Discovery of methylated circulating DNA biomarkers for comprehensive non‐invasive monitoring of treatment response in metastatic colorectal cancer. Gut. 2018;67(11):1995‐2005. 28982739 10.1136/gutjnl-2016-313372 PMC5897187

[124] Church TR , Wandell M , Lofton‐Day C , et al. Prospective evaluation of methylated SEPT9 in plasma for detection of asymptomatic colorectal cancer. Gut. 2014;63(2):317‐325. 23408352 10.1136/gutjnl-2012-304149 PMC3913123

[125] Liang L , Zhang Y , Li C , et al. Plasma cfDNA methylation markers for the detection and prognosis of ovarian cancer. eBioMedicine. 2022;83:104222. 35973389 10.1016/j.ebiom.2022.104222 PMC9396542

[126] Singh A , Gupta S , Badarukhiya JA , Sachan M . Detection of aberrant methylation of HOXA9 and HIC1 through multiplex MethyLight assay in serum DNA for the early detection of epithelial ovarian cancer. Int J Cancer. 2020;147(6):1740‐1752. 32191343 10.1002/ijc.32984

[127] Wang B , Yu L , Yang GZ , Luo X , Huang L . Application of multiplex nested methylated specific PCR in early diagnosis of epithelial ovarian cancer. Asian Pac J Cancer Prev APJCP. 2015;16(7):3003‐3007. 25854397 10.7314/apjcp.2015.16.7.3003

[128] Klein EA , Richards D , Cohn A , et al. Clinical validation of a targeted methylation‐based multi‐cancer early detection test using an independent validation set. Ann Oncol. 2021;32(9):1167‐1177. 34176681 10.1016/j.annonc.2021.05.806

[129] Chen X , Gole J , Gore A , et al. Non‐invasive early detection of cancer four years before conventional diagnosis using a blood test. Nat Commun. 2020;11:3475. 32694610 10.1038/s41467-020-17316-z PMC7374162

[130] Liu MC , Oxnard GR , Klein EA , Swanton C , Seiden MV . Sensitive and specific multi‐cancer detection and localization using methylation signatures in cell‐free DNA. Ann Oncol Off J Eur Soc Med Oncol. 2020;31(6):745‐759. 10.1016/j.annonc.2020.02.011 PMC8274402 33506766

[131] Mahon KL , Qu W , Devaney J , et al. Methylated Glutathione S‐transferase 1 (mGSTP1) is a potential plasma free DNA epigenetic marker of prognosis and response to chemotherapy in castrate‐resistant prostate cancer. Br J Cancer. 2014;111(9):1802‐1809. 25144624 10.1038/bjc.2014.463 PMC4453725

[132] Gordevičius J , Kriščiūnas A , Groot DE , et al. Cell‐free DNA modification dynamics in abiraterone acetate‐treated prostate cancer patients. Clin Cancer Res. 2018;24(14):3317‐3324. 29615462 10.1158/1078-0432.CCR-18-0101

[133] Rusan M , Andersen RF , Jakobsen A , Steffensen KD . Circulating HOXA9‐methylated tumour DNA: a novel biomarker of response to poly (ADP‐ribose) polymerase inhibition in BRCA‐mutated epithelial ovarian cancer. Eur J Cancer. 2020;125:121‐129. 31865042 10.1016/j.ejca.2019.11.012

[134] Dulaimi E , Hillinck J , de Caceres II , Al‐Saleem T , Cairns P . Tumor suppressor gene promoter hypermethylation in serum of breast cancer patients. Clin Cancer Res. 2004;10(18):6189‐6193. 15448006 10.1158/1078-0432.CCR-04-0597

[135] Hoque MO , Feng Q , Toure P , et al. Detection of aberrant methylation of four genes in plasma DNA for the detection of breast cancer. J Clin Oncol. 2016;24(26):4262‐9. 10.1200/JCO.2005.01.3516 16908936

[136] Tang Q , Cheng J , Cao X , Surowy H , Burwinkel B . Blood‐based DNA methylation as biomarker for breast cancer: a systematic review. Clin Epigenetics. 2016;8:115. 27895805 10.1186/s13148-016-0282-6 PMC5109688

[137] Boldrin E , Curtarello M , Dallan M , et al. Detection of LINE‐1 hypomethylation in cfDNA of esophageal adenocarcinoma patients. Int J Mol Sci. 2020;21(4):1547. 32102481 10.3390/ijms21041547 PMC7073170

[138] Ponomaryova AA , Rykova EY , Gervas PA , Cherdyntseva NV , Mamedov IZ , Azhikina TL . Aberrant methylation of LINE‐1 transposable elements: a search for cancer biomarkers. Cells. 2020;9(9):2017. 32887319 10.3390/cells9092017 PMC7563416

[139] Gainetdinov IV , KYu Kapitskaya , EYu Rykova , et al. Hypomethylation of human‐specific family of LINE‐1 retrotransposons in circulating DNA of lung cancer patients. Lung Cancer. 2016;99:127‐130. 27565927 10.1016/j.lungcan.2016.07.005

[140] Ponomaryova AA , Cherdyntseva NV , Bondar AA , et al. Dynamics of LINE‐1 retrotransposon methylation levels in circulating DNA from lung cancer patients undergoing antitumor therapy. Mol Biol (Mosk). 2017;51(4):622‐628. 28900080 10.7868/S0026898417040140

[141] Tahiliani M , Koh KP , Shen Y , et al. Conversion of 5‐methylcytosine to 5‐hydroxymethylcytosine in mammalian DNA by MLL partner TET1. Science. 2009;324(5929):930‐935. 19372391 10.1126/science.1170116 PMC2715015

[142] He YF , Li BZ , Li Z , et al. Tet‐mediated formation of 5‐carboxylcytosine and its excision by TDG in mammalian DNA. Science. 2011;333(6047):1303‐1307. 21817016 10.1126/science.1210944 PMC3462231

[143] Branco MR , Ficz G , Reik W . Uncovering the role of 5‐hydroxymethylcytosine in the epigenome. Nat Rev Genet. 2012;13(1):7‐13. 10.1038/nrg3080 22083101

[144] Zeng C , Stroup EK , Zhang Z , Chiu BCH , Zhang W . Towards precision medicine: advances in 5‐hydroxymethylcytosine cancer biomarker discovery in liquid biopsy. Cancer Commun. 2019;39(1):12. 10.1186/s40880-019-0356-x PMC6440138 30922396

[145] Sharma M , Verma RK , Kumar S , Kumar V . Computational challenges in detection of cancer using cell‐free DNA methylation. Comput Struct Biotechnol J. 2021;20:26‐39. 34976309 10.1016/j.csbj.2021.12.001 PMC8669313

[146] Song CX , Szulwach KE , Fu Y , et al. Selective chemical labeling reveals the genome‐wide distribution of 5‐hydroxymethylcytosine. Nat Biotechnol. 2011;29(1):68‐72. 21151123 10.1038/nbt.1732 PMC3107705

[147] Mooijman D , Dey SS , Boisset JC , Crosetto N , Van Oudenaarden A . Single‐cell 5hmC sequencing reveals chromosome‐wide cell‐to‐cell variability and enables lineage reconstruction. Nat Biotechnol. 2016;34(8):852‐856. 27347753 10.1038/nbt.3598

[148] Cai J , Chen L , Zhang Z , et al. Genome‐wide mapping of 5‐hydroxymethylcytosines in circulating cell‐free DNA as a non‐invasive approach for early detection of hepatocellular carcinoma. Gut. 2019;68(12):2195‐2205. 31358576 10.1136/gutjnl-2019-318882 PMC6872444

[149] Huang Y , Rao A . Connections between TET proteins and aberrant DNA modification in cancer. Trends Genet. 2014;30(10):464‐474. 25132561 10.1016/j.tig.2014.07.005 PMC4337960

[150] Song CX , Yin S , Ma L , et al. 5‐Hydroxymethylcytosine signatures in cell‐free DNA provide information about tumor types and stages. Cell Res. 2017;27(10):1231‐1242. 28820176 10.1038/cr.2017.106 PMC5630676

[151] Guler GD , Ning Y , Ku CJ , et al. Detection of early stage pancreatic cancer using 5‐hydroxymethylcytosine signatures in circulating cell free DNA. Nat Commun. 2020;11(1):5270. 33077732 10.1038/s41467-020-18965-w PMC7572413

[152] Haan D , Bergamaschi A , Friedl V , et al. Epigenomic blood‐based early detection of pancreatic cancer employing cell‐free DNA. Clin Gastroenterol Hepatol. 2023:S1542356523002240. 10.1016/j.cgh.2023.03.016 36967102

[153] Li W , Zhang X , Lu X , et al. 5‐Hydroxymethylcytosine signatures in circulating cell‐free DNA as diagnostic biomarkers for human cancers. Cell Res. 2017;27(10):1243‐1257. 28925386 10.1038/cr.2017.121 PMC5630683

[154] Millán‐Zambrano G , Burton A , Bannister AJ , Schneider R . Histone post‐translational modifications—cause and consequence of genome function. Nat Rev Genet. 2022;23(9):563‐580. 35338361 10.1038/s41576-022-00468-7

[155] McAnena P , Brown JAL , Kerin MJ . Circulating nucleosomes and nucleosome modifications as biomarkers in cancer. Cancers. 2017;9(1):5. 28075351 10.3390/cancers9010005 PMC5295776

[156] Noberini R , Osti D , Miccolo C , et al. Extensive and systematic rewiring of histone post‐translational modifications in cancer model systems. Nucleic Acids Res. 2018;46(8):3817‐3832. 29618087 10.1093/nar/gky224 PMC5934616

[157] Bates SE . Epigenetic therapies for cancer. Longo DL, ed. N Engl J Med. 2020;383(7):650‐663. 32786190 10.1056/NEJMra1805035

[158] Lichtenstein AV , Melkonyan HS , Tomei LD , Umansky SR . Circulating nucleic acids and apoptosis. Ann N Y Acad Sci. 2001;945(1):239‐249. 11708486 10.1111/j.1749-6632.2001.tb03892.x

[159] Snyder MW , Kircher M , Hill AJ , Daza RM , Shendure J . Cell‐free DNA comprises an in vivo nucleosome footprint that informs its tissues‐of‐origin. Cell. 2016;164(0):57‐68. 26771485 10.1016/j.cell.2015.11.050 PMC4715266

[160] Sozzi G , Roz L , Conte D , et al. Effects of prolonged storage of whole plasma or isolated plasma DNA on the results of circulating DNA quantification assays. JNCI J Natl Cancer Inst. 2005;97(24):1848‐1850. 16368947 10.1093/jnci/dji432

[161] Holdenrieder S , Pawel JV , Nagel D , Stieber P . Long‐term stability of circulating nucleosomes in serum. Anticancer Res. 2010;30(5):1613‐1615. 20592350

[162] Deligezer U , Akisik EE , Erten N , Dalay N . Sequence‐specific histone methylation is detectable on circulating nucleosomes in plasma. Clin Chem. 2008;54(7):1125‐1131. 18487283 10.1373/clinchem.2007.101766

[163] Bauden M , Pamart D , Ansari D , et al. Circulating nucleosomes as epigenetic biomarkers in pancreatic cancer. Clin Epigenetics. 2015;7:106. 26451166 10.1186/s13148-015-0139-4 PMC4597435

[164] Rahier JF , Druez A , Faugeras L , et al. Circulating nucleosomes as new blood‐based biomarkers for detection of colorectal cancer. Clin Epigenetics. 2017;9(1):53. 28515797 10.1186/s13148-017-0351-5 PMC5433015

[165] Sadeh R , Sharkia I , Fialkoff G , et al. ChIP‐seq of plasma cell‐free nucleosomes identifies gene expression programs of the cells‐of‐origin. Nat Biotechnol. 2021;39(5):586‐598. 33432199 10.1038/s41587-020-00775-6 PMC7610786

[166] Fedyuk V , Shema E , Erez N . Multiplexed single‐molecule epigenetic analysis of plasma‐isolated nucleosomes for cancer diagnostics. *Nat Biotechnol* . 2023;41:212–221. 10.1038/s41587-022-01447-3 36076083

[167] Guttman M , Amit I , Garber M , et al. Chromatin signature reveals over a thousand highly conserved large non‐coding RNAs in mammals. Nature. 2009;458(7235):223‐227. 19182780 10.1038/nature07672 PMC2754849

[168] Shi T , Gao G , Cao Y . Long noncoding RNAs as novel biomarkers have a promising future in cancer diagnostics. Dis Markers. 2016;2016:1‐10. 10.1155/2016/9085195 PMC4842029 27143813

[169] Gao Y , Wang JW , Ren JY , et al. Long noncoding RNAs in gastric cancer: from molecular dissection to clinical application. World J Gastroenterol. 2020;26(24):3401‐3412. 32655264 10.3748/wjg.v26.i24.3401 PMC7327794

[170] Volovat SR , Volovat C , Hordila I , et al. MiRNA and LncRNA as potential biomarkers in triple‐negative breast cancer: a review. Front Oncol. 2020;10:526850. 33330019 10.3389/fonc.2020.526850 PMC7716774

[171] Toden S , Goel A . Non‐coding RNAs as liquid biopsy biomarkers in cancer. Br J Cancer. 2022;126(3):351‐360. 35013579 10.1038/s41416-021-01672-8 PMC8810986

[172] Lee RC , Feinbaum RL , Ambros V . The C. elegans heterochronic gene lin‐4 encodes small RNAs with antisense complementarity to lin‐14. Cell. 1993;75(5):843‐854. 8252621 10.1016/0092-8674(93)90529-y

[173] Singh R , Ramasubramanian B , Kanji S , Chakraborty AR , Haque SJ , Chakravarti A . Circulating microRNAs in cancer: hope or hype? Cancer Lett. 2016;381(1):113‐121. 27471105 10.1016/j.canlet.2016.07.002

[174] Hamam R , Hamam D , Alsaleh KA , et al. Circulating microRNAs in breast cancer: novel diagnostic and prognostic biomarkers. Cell Death Dis. 2017;8(9):e3045. 28880270 10.1038/cddis.2017.440 PMC5636984

[175] Valihrach L , Androvic P , Kubista M . Circulating miRNA analysis for cancer diagnostics and therapy. Mol Aspects Med. 2020;72:100825. 31635843 10.1016/j.mam.2019.10.002

[176] Wang H , Peng R , Wang J , Qin Z , Xue L . Circulating microRNAs as potential cancer biomarkers: the advantage and disadvantage. Clin Epigenetics. 2018;10:59. 29713393 10.1186/s13148-018-0492-1 PMC5913875

[177] de Ronde MWJ , Ruijter JM , Moerland PD , Creemers EE , Pinto‐Sietsma SJ . Study design and qPCR data analysis guidelines for reliable circulating miRNA biomarker experiments: a review. Clin Chem. 2018;64(9):1308‐1318. 29903876 10.1373/clinchem.2017.285288

[178] Coenen‐Stass AML , Pauwels MJ , Hanson B , et al. Extracellular microRNAs exhibit sequence‐dependent stability and cellular release kinetics. RNA Biol. 2019;16(5):696‐706. 30836828 10.1080/15476286.2019.1582956 PMC6546368

[179] Lawrie CH , Gal S , Dunlop M , et al. Detection of elevated levels of tumour‐associated microRNAs in serum of patients with diffuse large B‐cell lymphoma. Br J Haematol. 2008;141(5):672‐5. 10.1111/j.1365-2141.2008.07077.x 18318758

[180] Mitchell PS , Parkin RK , Kroh EM , et al. Circulating microRNAs as stable blood‐based markers for cancer detection. Proc Natl Acad Sci USA. 2008;105(30):10513‐10518. 18663219 10.1073/pnas.0804549105 PMC2492472

[181] Miyoshi J , Zhu Z , Luo A , et al. A microRNA‐based liquid biopsy signature for the early detection of esophageal squamous cell carcinoma: a retrospective, prospective and multicenter study. Mol Cancer. 2022;21:44. 35148754 10.1186/s12943-022-01507-x PMC8832722

[182] Taylor DD , Gercel‐Taylor C . MicroRNA signatures of tumor‐derived exosomes as diagnostic biomarkers of ovarian cancer. Gynecol Oncol. 2008;110(1):13‐21. 18589210 10.1016/j.ygyno.2008.04.033

[183] Chung YW , Bae HS , Song JY , et al. Detection of microRNA as novel biomarkers of epithelial ovarian cancer from the serum of ovarian cancer patient. Int J Gynecol Cancer. 2013;23(4). 10.1097/IGC.0b013e31828c166d 23542579

[184] Vigneron N , Vernon M , Meryet‐Figuière M , et al. Predictive relevance of circulating miR‐622 in patients with newly diagnosed and recurrent high‐grade serous ovarian carcinoma. Clin Chem. 2020;66(2):352‐362. 32040573 10.1093/clinchem/hvz013

[185] Zhu W , Zhou K , Zha Y , et al. Diagnostic value of serum miR‐182, miR‐183, miR‐210, and miR‐126 levels in patients with early‐stage non‐small cell lung cancer. PLoS ONE. 2016;11(4):e0153046. 27093275 10.1371/journal.pone.0153046 PMC4836744

[186] Lampignano R , Kloten V , Krahn T , Schlange T . Integrating circulating miRNA analysis in the clinical management of lung cancer: present or future? Mol Aspects Med. 2020;72:100844. 31959359 10.1016/j.mam.2020.100844

[187] Li X , Zhou J , Xiao M , et al. Uncovering the subtype‐specific molecular characteristics of breast cancer by multiomics analysis of prognosis‐associated genes, driver genes, signaling pathways, and immune activity. Front Cell Dev Biol. 2021;9:689028. 34277633 10.3389/fcell.2021.689028 PMC8280810

[188] Shin VY , Siu JM , Cheuk I , Ng EKO , Kwong A . Circulating cell‐free miRNAs as biomarker for triple‐negative breast cancer. Br J Cancer. 2015;112(11):1751‐1759. 25906045 10.1038/bjc.2015.143 PMC4647231

[189] Eichelser C , Stückrath I , Müller V , et al. Increased serum levels of circulating exosomal microRNA‐373 in receptor‐negative breast cancer patients. Oncotarget. 2014;5(20):9650‐9663. 25333260 10.18632/oncotarget.2520 PMC4259427

[190] Fan T , Mao Y , Sun Q , et al. Branched rolling circle amplification method for measuring serum circulating microRNA levels for early breast cancer detection. Cancer Sci. 2018;109(9):2897‐2906. 29981251 10.1111/cas.13725 PMC6125458

[191] Hansen TF , Carlsen AL , Heegaard NHH , Sørensen FB , Jakobsen A . Changes in circulating microRNA‐126 during treatment with chemotherapy and bevacizumab predicts treatment response in patients with metastatic colorectal cancer. Br J Cancer. 2015;112(4):624‐629. 25584492 10.1038/bjc.2014.652 PMC4333496

[192] Summerer I , Niyazi M , Unger K , et al. Changes in circulating microRNAs after radiochemotherapy in head and neck cancer patients. Radiat Oncol Lond Engl. 2013;8:296. 10.1186/1748-717X-8-296 PMC3882107 24373621

[193] Jenike AE , Halushka MK . miR‐21: a non‐specific biomarker of all maladies. Biomark Res. 2021;9(1):18. 33712063 10.1186/s40364-021-00272-1 PMC7953557

[194] Decruyenaere P , Offner F , Vandesompele J . Circulating RNA biomarkers in diffuse large B‐cell lymphoma: a systematic review. Exp Hematol Oncol. 2021;10:13. 33593440 10.1186/s40164-021-00208-3 PMC7885416

[195] Pardini B , Sabo AA , Birolo G , Calin GA . Noncoding RNAs in extracellular fluids as cancer biomarkers: the new frontier of liquid biopsies. Cancers. 2019;11(8):1170. 31416190 10.3390/cancers11081170 PMC6721601

[196] Nagasaka M , Uddin MH , Al‐Hallak MN , et al. Liquid biopsy for therapy monitoring in early‐stage non‐small cell lung cancer. Mol Cancer. 2021;20(1):82. 34074295 10.1186/s12943-021-01371-1 PMC8170728

[197] Rittenhouse H , Blase A , Shamel B , Schalken J , Groskopf J . The long and winding road to FDA approval of a novel prostate cancer test: our story. Clin Chem. 2013;59(1):32‐34. 23193060 10.1373/clinchem.2012.198739

[198] Lin DW , Newcomb LF , Brown EC , et al. Urinary TMPRSS2:eRG and PCA3 in an active surveillance cohort: results from a baseline analysis in the Canary Prostate Active Surveillance Study. Clin Cancer Res Off J Am Assoc Cancer Res. 2013;19(9):2442‐2450. 10.1158/1078-0432.CCR-12-3283 PMC3674574 23515404

[199] Leyten GHJM , Hessels D , Jannink SA , et al. Prospective multicentre evaluation of PCA3 and TMPRSS2‐ERG gene fusions as diagnostic and prognostic urinary biomarkers for prostate cancer. Eur Urol. 2014;65(3):534‐542. 23201468 10.1016/j.eururo.2012.11.014

[200] Newcomb LF , Zheng Y , Faino AV , et al. Performance of PCA3 and TMPRSS2:eRG urinary biomarkers in prediction of biopsy outcome in the Canary Prostate Active Surveillance Study (PASS). Prostate Cancer Prostatic Dis. 2019;22(3):438‐445. 30664734 10.1038/s41391-018-0124-z PMC6642858

[201] Zhao W , Liu Y , Zhang C , Duan C . Multiple roles of exosomal long noncoding RNAs in cancers. BioMed Res Int. 2019;2019:1‐12. 10.1155/2019/1460572 PMC6642753 31360701

[202] Mugoni V , Ciani Y , Nardella C , Demichelis F . Circulating RNAs in prostate cancer patients. Cancer Lett. 2022;524:57‐69. 34656688 10.1016/j.canlet.2021.10.011

[203] Dey Ghosh R , Guha Majumder S . Circulating long non‐coding RNAs could be the potential prognostic biomarker for liquid biopsy for the clinical management of oral squamous cell carcinoma. Cancers. 2022;14(22):5590. 36428681 10.3390/cancers14225590 PMC9688117

[204] Ko J , Baldassano SN , Loh PL , Kording K , Litt B , Issadore D . Machine learning to detect signatures of disease in liquid biopsies—a user's guide. Lab Chip. 2018;18(3):395‐405. 29192299 10.1039/c7lc00955k PMC5955608

[205] Liu Y , Siejka‐Zielińska P , Velikova G , et al. Bisulfite‐free direct detection of 5‐methylcytosine and 5‐hydroxymethylcytosine at base resolution. Nat Biotechnol. 2019;37(4):424‐429. 30804537 10.1038/s41587-019-0041-2

[206] Siejka‐Zielińska P , Cheng J , Jackson F , et al. Cell‐free DNA TAPS provides multimodal information for early cancer detection. Sci Adv. 2021;7(36):eabh0534. 34516908 10.1126/sciadv.abh0534 PMC8442905

[207] Cardinali B , Tasso R , Piccioli P , Ciferri MC , Quarto R , Del Mastro L . Circulating miRNAs in breast cancer diagnosis and prognosis. Cancers. 2022;14(9):2317. 35565446 10.3390/cancers14092317 PMC9101355

[208] Kourou K , Exarchos KP , Papaloukas C , Sakaloglou P , Exarchos T , Fotiadis DI . Applied machine learning in cancer research: a systematic review for patient diagnosis, classification and prognosis. Comput Struct Biotechnol J. 2021;19:5546‐5555. 34712399 10.1016/j.csbj.2021.10.006 PMC8523813

[209] Liu L , Chen X , Petinrin OO , et al. Machine learning protocols in early cancer detection based on liquid biopsy: a survey. Life. 2021;11(7):638. 34209249 10.3390/life11070638 PMC8308091

[210] Bahado‐Singh R , Vlachos KT , Aydas B , Gordevicius J , Radhakrishna U , Vishweswaraiah S . Precision oncology: artificial intelligence and DNA methylation analysis of circulating cell‐free DNA for lung cancer detection. Front Oncol. 2022;12:790645. 35600397 10.3389/fonc.2022.790645 PMC9114890

[211] Li W , Li Q , Kang S , et al. CancerDetector: ultrasensitive and non‐invasive cancer detection at the resolution of individual reads using cell‐free DNA methylation sequencing data. Nucleic Acids Res. 2018;46(15):e89. 29897492 10.1093/nar/gky423 PMC6125664

[212] Li J , Wei L , Zhang X , et al. DISMIR: d eep learning‐based noninvasive cancer detection by i ntegrating DNA s equence and methylation information of i ndividual cell‐free DNA reads. Brief Bioinform. 2021;22(6):bbab250. 34245239 10.1093/bib/bbab250 PMC8575022

[213] Yu F , Li K , Li S , et al. CFEA: a cell‐free epigenome atlas in human diseases. Nucleic Acids Res. 2020;48(D1):D40‐D44. 31428785 10.1093/nar/gkz715 PMC6943076

[214] Prensner JR , Rubin MA , Wei JT , Chinnaiyan AM . Beyond PSA: the next generation of prostate cancer biomarkers. Sci Transl Med. 2012;4(127):127rv3. 10.1126/scitranslmed.3003180 PMC3799996 22461644

[215] Schwartz LH , Seymour L , Litière S , et al. RECIST 1.1 – Standardisation and disease‐specific adaptations: perspectives from the RECIST Working Group. Eur J Cancer Oxf Engl 1990. 2016;62:138‐145. 10.1016/j.ejca.2016.03.082 PMC5737786 27237360

[216] Thierry AR , El Messaoudi S , Gahan PB , Anker P , Stroun M . Origins, structures, and functions of circulating DNA in oncology. Cancer Metastasis Rev. 2016;35(3):347‐376. 27392603 10.1007/s10555-016-9629-x PMC5035665

[217] Keller L , Belloum Y , Wikman H , Pantel K . Clinical relevance of blood‐based ctDNA analysis: mutation detection and beyond. Br J Cancer. 2021;124(2):345‐358. 32968207 10.1038/s41416-020-01047-5 PMC7852556

[218] Wen L , Li J , Guo H , et al. Genome‐scale detection of hypermethylated CpG islands in circulating cell‐free DNA of hepatocellular carcinoma patients. Cell Res. 2015;25(12):1376. 26620315 10.1038/cr.2015.141 PMC4670997

[219] D'Auria F , Rovere‐Querini P , Giazzon M , et al. Accumulation of plasma nucleosomes upon treatment with anti‐tumour necrosis factor‐alpha antibodies. J Intern Med. 2004;255(3):409‐418. 14871466 10.1111/j.1365-2796.2003.01298.x

[220] Ellinger J , El Kassem N , Heukamp LC , et al. Hypermethylation of cell‐free serum DNA indicates worse outcome in patients with bladder cancer. J Urol. 2008;179(1):346‐352. 18006010 10.1016/j.juro.2007.08.091

[221] Van den Ackerveken P , Lobbens A , Turatsinze JV , et al. A novel proteomics approach to epigenetic profiling of circulating nucleosomes. Sci Rep. 2021;11(1):7256. 33790358 10.1038/s41598-021-86630-3 PMC8012598

[222] Cavalier E , Guiot J , Lechner K , et al. Circulating nucleosomes as potential markers to monitor COVID‐19 disease progression. Front Mol Biosci. 2021;8:600881. 33816549 10.3389/fmolb.2021.600881 PMC8012533

